# Neuropsychiatric Symptoms of COVID-19 Explained by SARS-CoV-2 Proteins’ Mimicry of Human Protein Interactions

**DOI:** 10.3389/fnhum.2021.656313

**Published:** 2021-03-23

**Authors:** Hale Yapici-Eser, Yunus Emre Koroglu, Ozgur Oztop-Cakmak, Ozlem Keskin, Attila Gursoy, Yasemin Gursoy-Ozdemir

**Affiliations:** ^1^Department of Psychiatry, School of Medicine, Koç University, Istanbul, Turkey; ^2^Research Center for Translational Medicine, Koç University, Istanbul, Turkey; ^3^Graduate School of Sciences and Engineering, College of Engineering, Koç University, Istanbul, Turkey; ^4^Department of Neurology, School of Medicine, Koç University, Istanbul, Turkey; ^5^College of Engineering, Chemical and Biological Engineering, Koç University, Istanbul, Turkey; ^6^Department of Computer Science and Engineering, College of Engineering, Koç University, Istanbul, Turkey

**Keywords:** COVID-19, SARS-CoV-2, neuropsychiatric, delirium, mimicry, autoimmune

## Abstract

The first clinical symptoms focused on the presentation of coronavirus disease 2019 (COVID-19) have been respiratory failure, however, accumulating evidence also points to its presentation with neuropsychiatric symptoms, the exact mechanisms of which are not well known. By using a computational methodology, we aimed to explain the molecular paths of COVID-19 associated neuropsychiatric symptoms, based on the mimicry of the human protein interactions with SARS-CoV-2 proteins.

**Methods:** Available 11 of the 29 SARS-CoV-2 proteins’ structures have been extracted from Protein Data Bank. HMI-PRED (Host-Microbe Interaction PREDiction), a recently developed web server for structural PREDiction of protein-protein interactions (PPIs) between host and any microbial species, was used to find the “interface mimicry” through which the microbial proteins hijack host binding surfaces. Classification of the found interactions was conducted using the PANTHER Classification System.

**Results:** Predicted Human-SARS-CoV-2 protein interactions have been extensively compared with the literature. Based on the analysis of the molecular functions, cellular localizations and pathways related to human proteins, SARS-CoV-2 proteins are found to possibly interact with human proteins linked to synaptic vesicle trafficking, endocytosis, axonal transport, neurotransmission, growth factors, mitochondrial and blood-brain barrier elements, in addition to its peripheral interactions with proteins linked to thrombosis, inflammation and metabolic control.

**Conclusion:** SARS-CoV-2-human protein interactions may lead to the development of delirium, psychosis, seizures, encephalitis, stroke, sensory impairments, peripheral nerve diseases, and autoimmune disorders. Our findings are also supported by the previous in vivo and in vitro studies from other viruses. Further in vivo and in vitro studies using the proteins that are pointed here, could pave new targets both for avoiding and reversing neuropsychiatric presentations.

## Introduction

First identified in December 2019, coronavirus disease 2019 (COVID-19) has been announced as a pandemic and as of August 26th, 2020, more than 23 million confirmed cases, and more than 815.000 confirmed deaths around 216 countries have been announced^1^.

Accumulating evidence points to its presentation with neuropsychiatric symptoms. The patients may present with delirium due to unknown etiology (Alkeridy et al., 2020; Beach et al., 2020; Hosseini et al., 2020). A retrospective analysis of 214 patients diagnosed with COVID-19, revealed that 36.4% of patients experience a neurological symptom (Mao et al., 2020). In an analysis of 58 patients admitted to intensive care unit, agitation (69%) and cranial MRI lesions as either mini-strokes or decreased cerebral blood flow in 8 patients were reported (Helms et al., 2020). Higher incidence of embolism (Poissy et al., 2020) and risk for coagulation (Xiong et al., 2020), ischemic and hemorrhagic stroke (Beyrouti et al., 2020; Ellul et al., 2020; Gonzalez-Pinto et al., 2020; Paterson et al., 2020), confusion, encephalopathy, and seizures (Hosseini et al., 2020; Karimi et al., 2020), ataxia, headache, dizziness, and sensory impairment (Herman et al., 2020; Mao et al., 2020), cranial neuropathies (Dinkin et al., 2020), decreased smell function (Moein et al., 2020) and anosmia (Boscolo-Rizzo et al., 2020; Hopkins et al., 2020; Lechien et al., 2020; Pallanti, 2020), decreased taste dysfunction (Boscolo-Rizzo et al., 2020) and encephalitis (McAbee et al., 2020), and parainfectious peripheral nerve-related effects as Guillain Barre (Ottaviani et al., 2020; Padroni et al., 2020; Paterson et al., 2020; Sedaghat and Karimi, 2020; Toscano et al., 2020; Zhao et al., 2020), and Miller Fisher syndromes (Gutierrez-Ortiz et al., 2020) have also been reported as individual cases. Also presentation with stroke in 3 patients, resembling antiphospholipid antibody syndrome, showed an increased risk for neural autoimmunity (Zhang et al., 2020).

In a recent follow-up study of 125 patients in the United Kingdom, 57 ischaemic strokes, 9 intracerebral hemorrhages, 1 central nervous system (CNS) vasculitis, 39 altered mental status, 9 unspecified encephalopathies, 7 encephalitides, 21 new psychiatric diagnoses with altered mental state change, 10 new-onset psychosis, 6 neurocognitive (dementia-like) syndrome, and 4 affective disorder were reported (Varatharaj et al., 2020). A report of 43 patients followed due to COVID-19 diagnosis also replicated the high emergence of delirium, psychosis, and encephalitis (Paterson et al., 2020). Previous coronavirus infections also point to increased risk of depression, anxiety, and dysexecutive syndrome in the follow up of infected patients (Rogers et al., 2020; Varatharaj et al., 2020).

The observed neurological presentations could be a direct result of the SARS-CoV-2 virus or secondary to the hypoxic and inflammatory or embolic process triggered by SARS-CoV-2. Viruses have compact and small genomes, therefore they utilize the host cells’ machinery to establish critical functions by hi-jacking their host cellular mechanisms. They evolved to target and intervene host molecular mechanisms with their limited number of proteins. While doing this, viruses may mimic host protein interaction motifs and host proteins’ interaction pathways and hijack host cell’s proteins for their own needs while causing a loss or gain of function in host proteins (Elde and Malik, 2009; Halehalli and Nagarajaram, 2015; Guven-Maiorov et al., 2016). They target to alter signaling pathways to change the fate of the cells, activate inflammation-related pathways, and alter the host cell’s immune responses.

Some viruses as coronavirus may demonstrate a neurotrophic activity and they may infect the nervous tissue by a hematological route through various mechanisms such as infecting the epithelial cells and lymphocytes that can migrate to the brain, or a neural route as infection of olfactory neurons and retrograde transport, anterograde transport through axon bundles, transsynaptic passage and passing through the meningeal, blood brain barrier and blood-cerebrospinal fluid barrier (Ludlow et al., 2016; Desforges et al., 2019).

SARS-CoV virus, which affected several countries in 2003, was isolated from the postmortem brain of SARS-CoV patients (Ding et al., 2004; Xu et al., 2005) and was shown to enter the brain and infect multiple brain areas through olfactory nerves including the thalamus, cerebrum, and brainstem in a mice model (Netland et al., 2008). SARS-CoV proteins were reported to interact with claudin, myelin basic protein, and mitochondria in humans (Vojdani and Kharrazian, 2020). Human Coronavirus (HCoV) OC43 was also shown in the brain tissue of an immunocompromised infant who experienced encephalitis (Morfopoulou et al., 2016). HCoV RNA was previously detected in both gray matter and white matter of the postmortem brain tissue or cerebrospinal fluid of Multiple Sclerosis patients and HCoV had the ability to infect glial cells like astrocytes and microglia (Arbour et al., 2000) and caused necrosis of the neurons (Xu et al., 2005). Mice infected with HCoV-OC43 containing theY241H mutation in the spike glycoprotein exhibited hind-limb paralysis by leading to glutamate excitotoxicity (Brison et al., 2011, 2014).

For COVID-19, only one study so far investigated cerebrospinal fluid of SARS-CoV-2 positive patients presenting with stroke and subarachnoid hemorrhage and the result was negative (Al Saiegh et al., 2020). Most researchers focus on angiotensin converting enzyme 2 (ACE-2) receptor binding for the access of the SARS-CoV-2 to CNS (Calcagno et al., 2020), however, considering the large profile of neurological symptoms associated and different neurobiological pathways, and inconsistent findings of cerebrospinal fluid (CSF) positivity, it is necessary to search other candidate pathways for the coronavirus association with CNS.

Based on the clinical observations of neuropsychiatric manifestations of COVID-19 and previous knowledge on other coronavirus types’ neurotrophic effects, understanding virus-host interactions is critical to show the path of the virus to invade host cells and end up with CNS dysfunction. We hypothesized that mimicry of human protein interactions by SARS-CoV-2 proteins may help to understand the neurobiological pathways that underlie the neuropsychiatric manifestations of COVID-19. To do so, in this study, we first listed the available SARS-CoV-2 protein structures. Secondly, we utilized HMI-PRED tool to detect mimicry of human protein interactions by SARS-CoV-2 proteins, and lastly we classified these interactions for the molecular paths of COVID-19 associated neuropsychiatric symptoms. Our purpose was also to generate a list of protein targets that can guide researchers for further studies in in vivo and in vitro research.

## Methods

**FIGURE 1:**
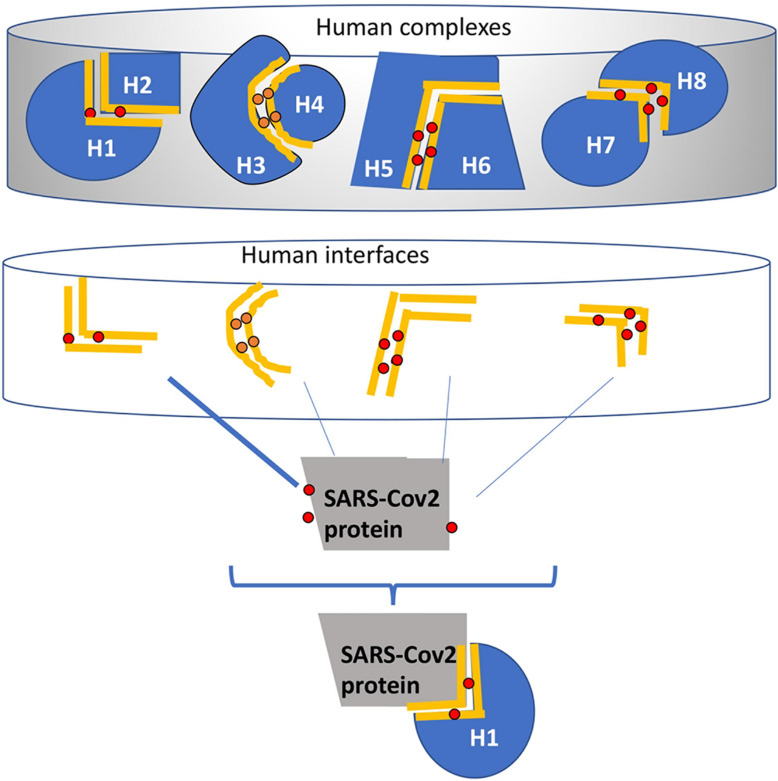
A schematic example of the HMI-Pred (human-virus protein interaction prediction) method. The initial set (Upper part) is all available structures of human protein-protein complexes extracted from Protein Data Bank. Interfaces that correspond to the binding sites on these complexes are extracted and form the template dataset (middle part). The red dots are the critical residues (hotspots) on these interfaces found by HotPoint (Tuncbag et al., 2010; Cukuroglu et al., 2012). The gray protein is the SARS-CoV-2 structure. In this example, the surface of the SARS protein has similar regions to the one side of the human interfaces (i.e., H2 side of the H1-H2 interface). The HMI_Pred algorithm assumes that since H1 forms a complex with H2 and SARS is similar to H2, SARS protein can complement and interact with H1. The same hotspots are found at the same location as the new complex formed between the SARS-CoV-2 and human proteins.

This study used a four step approach. At the first step, all known structures of SARS-CoV-2 proteins by May 2020, have been extracted from Protein Data Bank (PDB). The list of proteins and their known functions are listed in below ‘SARS-CoV-2 proteins’ section. Secondly, HMI_Pred is used to find the possible interactions between SARS-CoV-2 and human proteins based on binding site mimicry. This algorithm requires the structures of SARS-CoV-2 and human proteins (Figure 1). Detailed explanation of HMI-PRED tool is given in ‘HMI_PRED Algorithm’ section. Thirdly, the list of human proteins predicted to interact with SARS-CoV-2 was loaded to PANTHER Classification System version 15.0 released on 2020-02-14^2^ (Mi et al., 2019), which is a comprehensive classification system of the whole genes and proteins, where data is presented for locations, pathways, and reactome, in addition to a functional classification list (Section ‘Classification of the found interactions’). PANTHER was developed in 2003 and then continuously updated for the needs of researchers. Fourth, the list of involved proteins in each category has been investigated and additional proteins that could take part in these pathways and that could be responsible for the neuropsychiatric representations of SARS-CoV-2 have been added by the researchers, based on the lists presented by the PANTHER analysis, authors’ knowledge and literature search for the direct and indirect impact on the CNS, to generate the hypothesis. As most of the molecules are shared between different neurotransmitters’ pathways, we grouped these proteins into synapse and neurotransmission related molecules, as well as pathways for growth factors, mitochondria, coagulation and hemostasis, blood-brain barrier elements and metabolic pathways.

### SARS-CoV-2 Proteins

First, the SARS-CoV-2 proteome has been investigated for finding their structures. Its proteome has a total of 29 proteins consisting of 16 nonstructural (NSP), 4 structural proteins (SP), 9 open reading frames (ORF) (Gordon et al., 2020). Four structural proteins are spike (S), membrane (M), envelope (E), and nucleocapsid (N) proteins. Spike (S) is a glycoprotein that forms homotrimers protruding from the virus capsid surface (Walls et al., 2020). It is responsible for the recognition and binding of the virus to the host cell ACE-2 receptor. It also mediates the entry into the host cell. The envelope (E) protein of SARS-CoV-2 oligomerizes to create an ion channel (Verdia-Baguena et al., 2012). It is thought to have several roles in viral pathogenesis including viral assembly (Lim and Liu, 2001), and virion release (Ruch and Machamer, 2012). M is an integral membrane glycoprotein, it is one of the main components of the envelope mainly functioning in viral assembly (Neuman et al., 2011). This protein interacts with the N protein to encapsulate the RNA genome (Siu et al., 2008). The nucleocapsid (N) is a phosphoprotein that takes part in stability (Grunewald et al., 2018).

Non-structural protein (NSP) genes constitute almost 60% of the viral genome and are translated into a large overlapping polyprotein 1a (NSP1-NSP11) and 1b (NSP12-NSP16). These proteins are essential for viral replication and translation. NSPs constitute of polymerases, helicases, exonucleases, and papain-like and chymotrypsin-like proteases. Nonstructural protein 1 (NSP1) might be a potent inhibitor of host gene expression (Yoshimoto, 2020). In SARS-CoV, it inactivates translation by binding to the 40S ribosome of the host cell, leading to host mRNA degradation selectively (Huang et al., 2011). In SARS-CoV, NSP2 interacts with two host proteins: prohibitin 1 and prohibitin 2 (PHB1 and PHB2) (Cornillez-Ty et al., 2009). Prohibitins take part in cell cycle progression, migration, differentiation, apoptosis, and mitochondrial biogenesis. Therefore, NSP2 might be important to alter the host cell environment. NSP3 is the papain-like proteinase which includes ssRNA binding, ADPr binding, G-quadruplex binding, protease (papain-like protease), and NSP4 binding, and transmembrane domains. NSP4 might have a membrane arrangement function in SARS-CoV and is essential for viral replication. NSP6 was shown to induce membrane vesicles. Nsp3-Nsp4-Nsp6 form a complex involved in viral replication (Angelini et al., 2013). NSP5 is the main protease (3C-like). It cleaves 11 distinct sites to yield mature and intermediate nonstructural proteins (NSPs) in MERS (Tomar et al., 2015). The Nsp7-Nsp8 complex is part of the RNA polymerase. This NSP7-NSP8 complex then binds to NSP12 which is the RNA-dependent RNA polymerase (Gordon et al., 2020). NSP8 protein alone may also complex with NSP12, forming the RNA polymerase complex. NSP9 has an ssRNA binding domain and might function in viral replication (Egloff et al., 2004). NSP10 interacts with NSP14 for its stimulation and NSP16 which are methyltransferases, in SARS-CoV (Ma et al., 2015; Wang et al., 2015). NSP13 is a helicase that unwinds duplex RNA, it also acts as a triphosphatase. NSP12 binds to NSP13 increasing its helicase activity (Jia et al., 2019). NSP14 has an exoribonuclease activity and N7-methyltransferase activity (Ivanov et al., 2004). NSP15 of SARS-CoV was shown as an endoribonuclease cleaving RNA at uridylates (Bhardwaj et al., 2006). It can specifically target and degrade the viral polyuridine sequences to hide the virus from the host immune system (Hackbart et al., 2020). NSP16 is a methyltransferase also acting in mRNA translation, and preventing the viral RNA from being recognized by host immunity. SARS-CoV-2 also comprises nine viral accessory proteins that form complexes with the structural proteins. SARS-CoV’s ORF3a protein is an ion channel protein involved in NLRP3 inflammasome activation. ORF6 is believed to be involved in virus-induced apoptosis. It interacts with NSP8. SARS-CoV ORF7a is an accessory protein that is transmembrane (Kumar et al., 2007). Other accessory proteins do not have assigned functions yet.

### HMI_PRED Algorithm

Host-Microbe Interaction PREDiction(HMI-PRED) (Guven-Maiorov et al., 2020) is a recently developed web server for structural PREDiction of protein-protein interactions (PPIs) between host and any microbial species, including bacteria, viruses, fungi, and protozoa. HMI-PRED is a modified version of our PRISM server (Tuncbag et al., 2011; Baspinar et al., 2014). HMI relies on “interface mimicry” through which the microbial proteins hijack host binding surfaces. Interface mimicry is defined as the structural similarity of the protein-protein binding sites and conservation of at least one hotspot residue (critical residues for interaction, if mutated the interaction is disrupted). Given the structure of a microbial protein of interest, HMI-PRED will provide the list of host-microbe interactions together with structural models of these potential host-microbe interactions (HMI). HMI_PRED is available at https://interactome.ku.edu.tr/hmi/. Figure 1 describes how HMI-PRED works. In the upper panel, there are the human protein-protein complexes derived from known PDB (Berman et al., 2000). This example figure displays only four complexes, in the HMI-Pred total number of human complexes (as of 2019) is around 53,000. Then, interface regions between two human proteins are extracted in these complexes (middle panel). The final template interface set includes 17351 structurally non-redundant interfaces. The user input is a (or a set of) microbial protein structure(s). HMI-Pred structurally compares the surface of the microbial proteins to the 17351 human interfaces. HMI-Pred uses two thresholds: TMscore (Zhang and Skolnick, 2005) (for interface mimicry, taken as 0.25 in HMI-Pred, however, mostly interactions with a score of 0.4 or above are considered in our study) and Rosetta I_sc^3^ (representing the interface binding energy, taken as -5 or better in HMI-Pred).

In this study, the host is the human and the microbe is the SARS-CoV-2. 11 of the 29 SARS-CoV-2 proteins have structures available, the list of SARS-CoV-2 proteins, their functions, and the PDB IDs for available structures are given in Supplementary Table 1.

### Classification of the Found Interactions

All human proteins from HMI-Pred predictions (SARS-CoV-2 protein- human protein interactions) have been uploaded to panterdb, and a functional classification for homo sapiens has been generated. Two bar graphics have been produced for the molecular function and secondly, cellular components of the proteins by selecting PANTHER-GO-Slim ontology. In addition, the pathways associated with the proteins have been determined by selecting PANTHER-Pathway ontology. Pathways represented more than expected has been analyzed using PANTHER statistical overrepresentation in the Homo Sapiens all genes database with Fisher’s exact test with Bonferroni correction for multiple testing.

## Results

**FIGURE 2:**
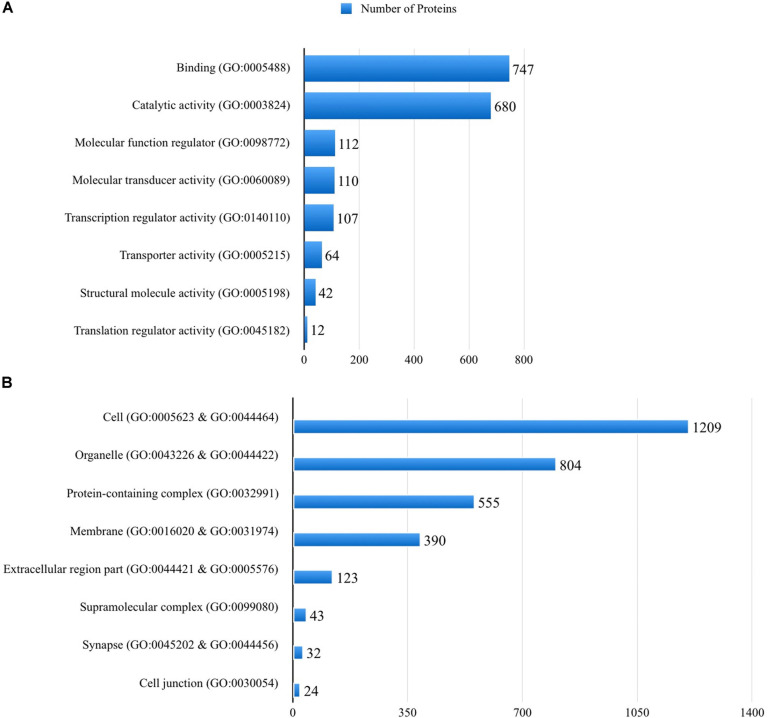
Molecular functions **(A)** and cellular distributions **(B)** of the human proteins that are mimicked by the SARS-CoV-2 proteins, according to the PANTHER classification system.

The full list of human proteins interacting with different protein structures of the SARS-CoV-2 is given in Supplementary Table 2. Of the 2256 genes that hit 1874 molecular functions, 33.1% showed a binding molecular activity, 30.1% was related to catalytic activity, whereas 2.8% was related to a transporter activity, based on the PANTHER classification (Figure 2A). When the cellular components of the human interactome with the SARS-CoV-2 proteins are analyzed, around 32 of the proteins were localized in the synapse, in addition to those localized to cell junction, cell part, and membranes of the cells (Figure 2B). When the pathways associated with the human proteins interacting with different protein structures of the SARS-CoV-2 are analyzed, 140 pathways were listed as given in Supplementary Table 3. Among these pathways, 33 pathways were found to be statistically overrepresented than expected in the SARS-CoV-2 proteins’ mimicry list of human protein interactions (Supplementary Table 4).

When the molecular functions, cellular localizations, and pathways related to human proteins are analyzed, it was found that SARS-CoV-2 may affect the brain both by a central effect and by indirect vascular and inflammatory effects.

### SARS-CoV-2 Mimics Synaptic Vesicle Priming, Fusion, Docking, and Endocytosis Related Proteins

**FIGURE 3:**
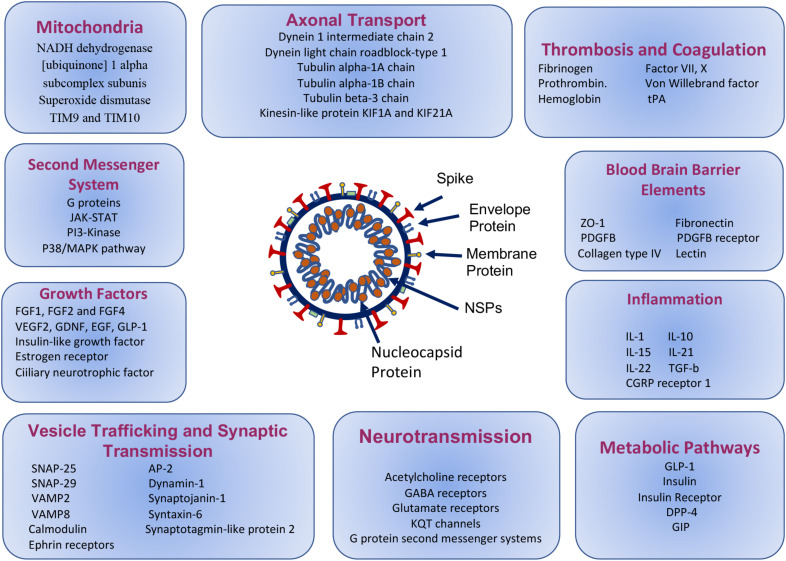
Molecular paths of COVID-19 associated neuropsychiatric symptoms, based on the mimicry of the selected human protein interactions with SARS-CoV-2 proteins.

**FIGURE 4:**
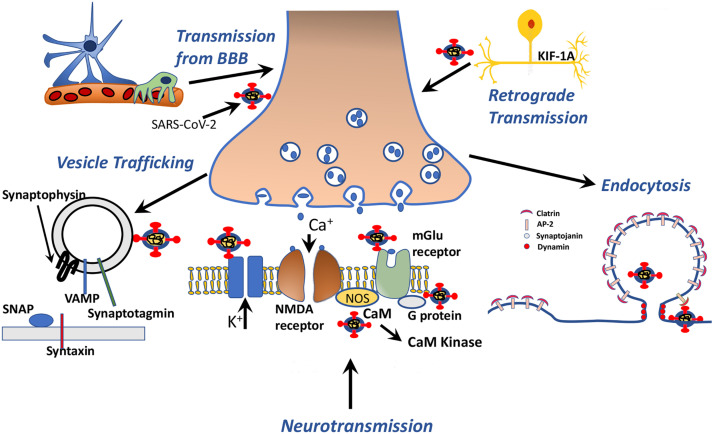
Schematic drawing representing SARS-CoV-2 protein-human proteins interactions associated with vesicle transport, neurotransmission, endocytosis and axonal transport.

The results are presented in Figures 3, 4 and Supplementary, Table 5. Among these molecules, AP-2, synaptojanin family, dynamins, and calcineurin related proteins mainly take part in endocytosis, whereas SNAP-25, SNAP-29, syntaxin, and VAMP2 take part in vesicle trafficking (Turner et al., 1999). AP-2 interactions may specifically be mimicked by nucleocapsid, spike, and NSP7 of SARS-CoV-2. SNAP-25 and SNAP-29, VAMP2 and VAMP8, which are all strongly mimicked by SARS-CoV-2 proteins, are also shared molecules for 5-HT, glutamate, acetylcholine, adrenaline, and noradrenaline, oxytocin neurotransmission, corticotropin-releasing factor receptor, and thyrotropin-releasing factor signaling, enkephalin release, GABA B receptor II signaling, opioid-prodynorphin/proenkephalin/proopiomelanocortin pathways (Supplementary Table 3 sublists) (Turner et al., 1999).

### SARS-CoV-2 Mimics Neurotransmission and Neuronal Excitability Related Proteins

**FIGURE 5:**
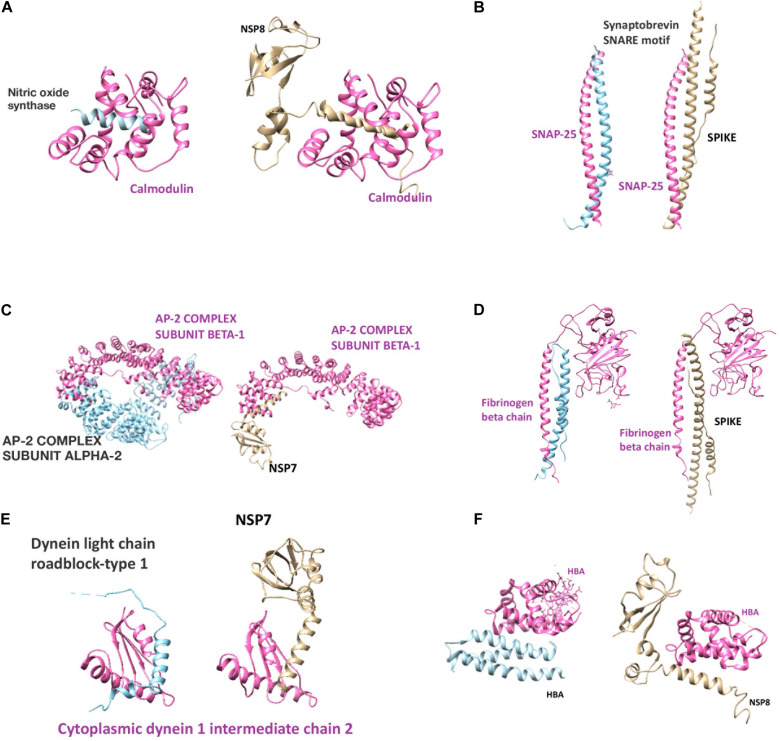
Putative structural models of SARS-CoV-2 human protein interactions. **(A–F)** Each subfigure represents the human protein-protein interaction and SARS-CoV-2 protein replacement of the human protein by mimicry. (Pink and blue colors demonstrate human proteins, gold color demonstrates SARS-CoV-2 protein).

The results are presented in Figure 4 and Supplementary Tables 2, 3, 6. Mainly spike, NSP7 and NSP8, also, some other NSP proteins mimic potassium voltage-gated channel subfamily KQT member 1 and 2, gamma-aminobutyric acid type A and B receptor subunits, glycine receptor subunits, metabotropic glutamate receptors, neuronal acetylcholine receptor subunits, dopamine D2 receptor, and adenosine receptor A2a. In addition to direct receptor interactions, SARS-CoV-2 proteins may mimic multiple proteins in both Gs, Gi, and Gq secondary messenger systems, which mediate metabotropic neurotransmission through both glutamatergic, adrenergic, histaminergic, cannabinoidergic, opioidergic, and serotonergic systems. In addition to histamine, serotonin, and acetylcholine synthesis and degradation (Supplementary Tables 2, 3, 6). Tryptophan 5-hydroxylase 2, which is an important enzyme for serotonin synthesis, also has a strong mimicry with the spike protein of SARS-CoV-2. Among these proteins, calmodulin-2 presents the strongest interaction with SARS-CoV-2 proteins (Supplementary Table 2). It is well established that increased intracellular calcium levels cause Nitric oxide synthase (NOS) enzyme activation through Ca-calmodulin. Both neuronal and endothelial NOS are activated with similar mechanisms. From our list, we have detected calmodulin as a strong interactor with viral protein (possible 3D model shown in Figure 5) and simulates NOS-Calmodulin binding (Aoyagi et al., 2003; Spratt et al., 2011).

### SARS-CoV-2 Mimics Major Growth Factors

The results are presented in Figure 3 and Supplementary Tables 2, 7, as FGF1, FGF2 and FGF4, VEGF2, GDNF, EGF, GLP-1, insulin-like growth factor, insulin and estrogen receptor, ciliary neurotrophic factor, that take a role in neuroprotection, cell growth and survival both in the brain and spinal cord neurons and astrocytes. These neurotrophic factors are coupled to Ras-MAPK pathways, PI3 kinase pathways, PLPCγ pathways, in addition to JAK-STAT signaling (Supplementary Table 3), whose associated proteins are not discussed specifically here.

### SARS-CoV-2 Mimics Mitochondrial Electron Transport and Mitochondrial Protein Import Related Proteins

The results are presented in Figure 3 and Supplementary Tables 2, 3, 8. SARS-CoV-2 proteins mimic human proteins that take a role in ATP synthesis, apoptosis, and oxidative stress where mitochondrial proteins are also involved (Supplementary Table 3). In addition to these pathways, SARS-CoV-2 proteins interact with many structural mitochondrial proteins and enzymes (Supplementary Table 8). Among these enzymes, NSP8 strongly mimics NADH dehydrogenase [ubiquinone] 1 beta subcomplex subunit 10 (Supplementary Table 2) interaction. Mitochondria also take an active role in glutamate-glutamine transmission by glutamate dehydrogenase which is also listed in the interactions, and this pathway is important in the lactate shuttle to neurons.

### SARS-CoV-2 Mimics Miscellaneous Proteins in the Brain

SARS-CoV-2 proteins mimic 17 proteins linked with Alzheimer’s disease- amyloid secretase pathway and 25 proteins linked with Alzheimer’s disease-presenilin pathway like beta-secretase, presenilin-1, amyloid-beta precursor and binding proteins, gamma-secretase subunit-2; 43 proteins linked to Parkinson’s disease pathways including alpha-synuclein (Supplementary Table 3), 26 axon guidance proteins (Supplementary Table 9), axonal transport proteins like cytoplasmic dynein 1 intermediate chain 2, dynein light chain roadblock-type 1, tubulin alpha-1A chain, tubulin alpha-1B chain, tubulin beta-3 chain, kinesin-like protein KIF1A and KIF21A (Figures 3, 4) and numerous other proteins linked to the central nervous system, as glia-derived nexin, glial fibrillary acidic protein, neural cell adhesion molecule 2 and survival motor neuron protein (Supplementary Table 2).

When the panther ontology database pathways that have been listed in Supplementary Table 3 are analyzed, it is found that SARS-CoV-2 may affect the brain through various indirect mechanisms such as thrombosis, vascular interactions involving BBB, and inflammation.

### SARS-CoV-2 Mimics Proteins in the Blood-Brain Barrier

**FIGURE 6:**
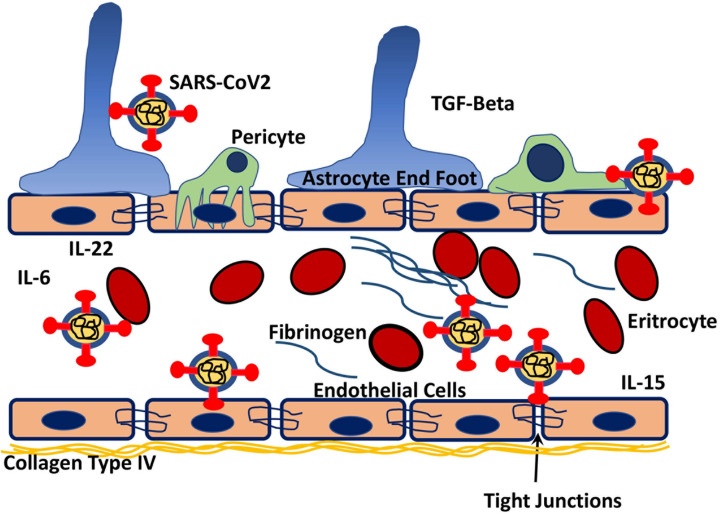
Schematic drawing of possible interactions of the virus with both blood coagulation elements as well as components of the blood-brain barrier. By interacting with tight junction protein ZO-1, extracellular matrix elements of BBB like fibronectin, collagen it might cause BBB dysfunction. Reacting and with possible blockage of PDGFβ released from endothelium and its receptor on pericytes may disrupt the integrity of BBB. Also interacting with fibrinogen and factor VII may lead to coagulopathy, and reaction with hemoglobin subunits may cause decreased oxygenation of the brain tissue due to decreased oxygen-binding capacity. Decreased binding of endothelial NOS to calmodulin hence less activation of NOS with decreased NO production may lead to disturbances in vascular reactions.

The results are presented in Figure 6 and Supplementary Tables 2, 10. We chose the vascular pathway from panther ontology which showed 188 possible interactions and from the HMI list, 21 proteins that may interact with several parts of the SARS-CoV-2 virus were chosen. For example both spike and NSP9 show mimicry for most prominent extracellular basement membrane protein collagen type IV. Interestingly, C-type lectin domain family members important in blood-brain barrier integrity may interact nearly all proteins of the virus (Kim Y. et al., 2020). Fibronectin mimicry goes with NSPs, GFAP with spike, solid carrier proteins with NSPs mainly, insulin receptor with spike and NSP9, integrins with several NSPs, laminin subunits with mostly spike, MMP13 with mainly 3CL-like protease, spike protein especially interacts with Zonula occludens-1, vimentin, protein S-100, laminin and collagen subunits, in addition to actin (Supplementary Tables 2, 10). PDGF beta and its receptor as well as eNOS calmodulin interaction were mimicked by viral proteins.

### SARS-CoV-2 Mimics Various Proteins That Increase the Vulnerability of Thrombosis

SARS-CoV-2 proteins mimic human proteins that are involved in blood coagulation and hemostasis. Both elements of prothrombotic and antithrombotic pathways show mimicry with viral particles. According to PANTHER ontogene pathways analysis, 24 proteins for blood coagulation and from HMI lists 20 manually chosen coagulation related proteins demonstrated molecular similarity. Proteins that are involved in coagulation are listed in Figure 3 and Supplementary Table 11 and schematically drawn in Figure 6. Fibrinogen and laminin may interact with spike protein. Interestingly hemoglobin subunit alpha, beta, and gamma have demonstrated molecular mimicry with viral proteins (Supplementary Tables 2, 11).

### SARS-CoV-2 Mimics Various Factors That Increase Peripheral Inflammatory Responses

The results are presented in Figure 3 and Supplementary Tables 2, 12. Some of these inflammatory proteins like IL-1, IL-10, IL-15, IL-21, and IL-22, may have roles in neural immune responses. Additionally, TGF-beta which is a strong stimulator of neural, pericyte, and endothelial immune responses may interact with different proteins of SARS-CoV-2. They may be involved in the inflammation mediated by chemokine and cytokine signaling pathways and T cell activation (Supplementary Table 3). Additionally, there is possible interaction and mimicry with CGRP receptor 1 and SARS-CoV-2.

### SARS-CoV-2 Mimics GLP-1, Insulin, DPP-4, and GIP

SARS-CoV-2 interacts with both insulin/IGF pathways (Supplementary Table 2) both by directly with insulin and its receptors and through its secondary effectors, also, it mimics GLP-1, GIP, and DPP-4 which affects GLP-1 levels. These molecules work together in the regulation of blood glucose levels (Foley, 2019). They have their receptors in the brain which will also be discussed for their direct effects.

## Discussion

Here, we demonstrate the human protein interactions of 11 SARS-CoV-2 proteins via a computational approach. SARS-CoV-2 proteins mimic the interactions of proteins on the blood-brain barrier, synapse, and glial structures. These findings support the fact that SARS-CoV-2 may be affecting the brain and leading neuropsychiatric symptoms.

In COVID-19 cases with neurological symptoms onset, the presence of viral particles in the frontal lobe, in small vesicles of endothelial cells and the neural cell bodies, confirming a transendothelial passage to the brain, was detected, while cerebrospinal fluid (CSF) SARS-CoV-2 RT-PCR was negative (Paniz-Mondolfi et al., 2020). Coronaviruses may interact with tight junction proteins as zonula occludens-1 for viral entry and pass to the brain by decreasing the integrity of tight junctions, in addition to spreading around the body through the bloodstream and invading the brain, as shown for other viruses (Torres-Flores and Arias, 2015). Olfactory epithelium and bulb were shown to be infected by the virus in postmortem investigation (Cantuti-Castelvetri et al., 2020). It is very well known that other viruses and some bacteria may modify blood brain barrier (BBB) components and induce inflammation leading to leakiness of BBB and passage to the brain and they may directly interact components of BBB like endothelial cells, pericytes, tight junction proteins, astrocyte end-feet, and matrix proteins. Viruses may interact with BBB endothelium through various receptors like insulin receptors, VEGF receptors, amino acid transport proteins. Particles may interact with adhesion molecules located on endothelium like integrins, also with tight junction proteins like ZO-1, claudin, and occludin as well. It was demonstrated that retroviruses leading to neural involvement like HIV and HTLV-1 can cause tight junction disintegration (Miller et al., 2012). Similarly, the Zika virus has been shown to alter BBB structure especially the tight junctions (Ljubin-Sternak et al., 2019). Additionally, the virus has an important interaction with ACE-2 receptors located on both endothelium as well as brain vascular pericytes. He et al. hypothesized that pericytes might be a key player in vascular complications of COVID-19 since they have a high-level expression of ACE-2 receptors (He et al., 2020). In addition to these findings, the virus may interact and block the effects of growth factors important in the maintenance of BBB, like PDGF beta and VEGF. We have also detected similarities of viral particles with extracellular matrix elements important in BBB structure and function like collagen, fibronectin, MMP inhibitors. Additionally, at the level of the endothelium, the interaction of viruses with calmodulin may block the activation of endothelial NOS leading to decreased physiological responses and functions of the endothelium (Cirino et al., 2003).

After invasion into the brain, SARS-CoV-2 may use endocytosis pathways through its interaction with AP-2, dynamin and other endocytosis related proteins and enter the neuron. Clathrin coated endocytosis is an evolutionary reserved process regulated by adaptor proteins as AP-2 and mediator proteins as dynamin and ephrin, which can be used by both the host cells and also viruses for entry (Rappoport et al., 2006). We found that both spike and other SARS-CoV-2 proteins may mimic endocytosis proteins as AP-2, dynamin-1, and calmodulin located in the synapse. Zika virus, which is found to affect the development of the brain significantly, was also found to interact with dynamin (Agrelli et al., 2019). Rabies virus, which is transmitted to humans after bite of the infected animals, and presenting with agitation, hydrophobia, and mortal consequences, uses dynamin-1 and AP-2 for endocytosis (Guo et al., 2019). Pharmacological targeting of the dynamin was found effective in avoiding the neural entry of the virus (Xu et al., 2015). Mutations in dynamin protein in humans and upregulation in rat models were found to be related to seizures (Li and Kavalali, 2017). Dynamin-1 is a large GTPase, that is used by many other viruses as adenovirus, hepatitis C virus, herpes simplex virus, influenza A virus, HIV-1, and others as listed in Sun and Tien (2013). Even though there are different types of dynamin and dynamin-like proteins, dynamin-1, whose interactive sites which were found to be mimicked by SARS-CoV-2 proteins, are more abundant in the brain (Sun and Tien, 2013).

Knowledge from the previous viruses may point to clathrin non-dependent cell entry pathways as through caveolin, flotillin-dependent or macropinocytosis can be used by SARS-CoV-2 in cell entry (Glebov, 2020). Our computational analysis also reveals a mimicry of binding motifs to endophilin-A1 and endophilin-A3 by the SARS-CoV-2 proteins (Supplementary Tables 2, 5). Endophilin both interacts with dynamin and β adrenergic receptors (Zhang et al., 2012). Endophilin may also be responsible for fast non-clathrin dependent, dynamin-dependent uptake of EGFR, VEGF, and PDGF like G protein-coupled receptors (Walpole and Grinstein, 2020).

Our analysis reveals that SARS-CoV-2 proteins may also mimic important proteins for synaptic vesicle cycling. After invading the neuron, it may use intracellular replication pathways for its reproduction and later use vesicular trafficking pathways through SNAP-29, SNAP-25, and other vesicle trafficking proteins (Li and Kavalali, 2017; Kadkova et al., 2019). By occupying the named proteins, it may also cause their loss of function due to occupation and lead to a disrupted vesicle trafficking that may indirectly affect 5-HT, glutamate, acetylcholine, adrenaline and noradrenaline, oxytocin neurotransmission, corticotropin-releasing factor receptor, and thyrotropin-releasing factor signaling, enkephalin release, GABA B receptor II signaling, and opioid-prodynorphin/proenkephalin/proopiomelanocortin pathways that use these molecules. Dysfunctions of these pathways may explain the observed reversible changes in mental state as delirium, hallucinations, and smell function in COVID-19 patients. This effect may also mimic the effect of botulinum toxin, which acts by entering the cytosol and cleaving the SNARE proteins (Rossetto et al., 2020). VAMP2, syntaxin-1, and SNAP-25 take a role in both spontaneous and active neurotransmitter release (Kadkova et al., 2019). Synaptotagmin on the vesicle membrane acts as a calcium sensor, and upon binding of Ca, it interacts with syntaxin-1 and SNAP-25 for the release of the neurotransmitters from the vesicles (Li and Kavalali, 2017).

VAMP2, tyrosine hydroxylase, and dopamine transporter are important molecules for dopamine neurotransmission in the striatum (Faustini et al., 2018). VAMP2 is also in interaction with alpha-synuclein and modulated alpha-synuclein aggregates (Faustini et al., 2018). VAMP2 increases insulin-stimulated GLUT4 translocation, which carries glucose into adipocytes, cardiac and skeletal muscles (Olson et al., 1997). A significant number of proteins in our analysis were linked with Parkinson’s disease pathways. Dopaminergic pathways could be more vulnerable to the infection of SARS-CoV-2 and possible neurodegeneration.

On the other hand, VAMP2 was shown on the T-lymphocytes and related to granule exocytosis in mice (Matti et al., 2013) and SNAP-29 in cytokine secretion from keratocytes (Meng et al., 2019). Therefore, it is possible in humans as well that interaction of SARS-CoV-2 with SNARE proteins could also have an immune function. Among the SNARE proteins, SNAP-29 may also affect both Golgi secretosome pathways, in addition to endosome/lysosome pathways (Kadkova et al., 2019). A previous study from China found that 22 (12.02%) of the 183 hospitalized children with clinically suspected acute encephalitis, were identified with a coronavirus infection and these patients expressed higher GM-CSF compared to coronavirus cases with respiratory symptoms (Li et al., 2016).

SARS-CoV-2 might also be using axonal transport proteins for its spread and infection in the neuron, as demonstrated by the mimicry of tubulin, KIF1A, KIF21A, and dynein. HCoV OC43 was also shown to use axonal transport pathways, which lead to neuron to neuron propagation and spread of the virus in the brain (Dube et al., 2018). In addition to accelerating the spread of the virus, it may disrupt the transport of major proteins and structures through the axon, as seen in the HIV-1 disruption of mitochondrial transport (Wang et al., 2017).

As for its postsynaptic effects, SARS-CoV-2 proteins interact both directly with receptors of GABA (mainly GABA B) and glutamate (mainly NMDA and metabotropic glutamate receptors) leading to change in the membrane resting-state potential and action potential, in addition to secondary messenger systems that may indirectly affect membrane resting-state potential, in addition to intracellular protein functioning. At the cellular level, there is a possibility that SARS-CoV-2 affects not only the neurons, but also it may affect astrocytes due to its affinity for Glial Fibrillary Acidic Protein (GFAP) which has a strong interaction potential.

SARS-CoV-2 may also use the receptors for entry to cells. Rabies virus was shown to use nicotinic acetylcholine receptors for entry into the brain (Guo et al., 2019). Especially K+, Na+, Cl- and Ca2+ channels are shown to be used by several viruses to enter the cell, and pharmaceutical targeting of viral entry through ion channels has shown promise in avoiding viral effects (Hover et al., 2017). On the other hand, by interacting with these ion channels, viruses may change neuronal firing through loss or gain of function of the ion channel, as seen in Rabies and Varicella Zoster infections (Hover et al., 2017). An in vitro study also pointed that SARS-CoV-2 is different from other coronavirus types for involving a unique S1/S2 cleavage site, which mimics a peptide on the human epithelial sodium channel α-subunit (ENaC-α) (Anand et al., 2020). The neuroinvasive potential of SARS-CoV-2 and its interaction with both ion channels and vesicle trafficking may also be effective in the peripheral nervous system and as suggested by Li Y. C. et al. (2020), may contribute to the respiratory failure by both invading to central respiration centers and peripheral nerves that innervate the lungs.

SARS-CoV-2 can affect the survival of the CNS cells through multiple pathways. An in vitro analysis of SARS-CoV-2 spike proteins with human tissue also found increased interactions with mitochondria-related proteins (Vojdani and Kharrazian, 2020). Viruses target mitochondrial dynamics as fusion and fission, change mitochondrial membrane potential and target various mechanisms of mitochondrial metabolism, as β-oxidation and the TCA cycle, for their virulence capacity as replication and persistence (Tiku et al., 2020). Dynamin-1-like protein is responsible for the fragmentation of mitochondria into smaller components, known as mitochondrial fission. When this mechanism is blocked, it is also shown to block cell to cell spread of some bacteria (Tiku et al., 2020). HIV-1 infected patients with neurobehavioral symptoms were also investigated for dynamin-like protein-1 expression in the frontal cortex and its expression level was found to be decreased around 50% (Fields et al., 2016). NADH dehydrogenase [ubiquinone] 1 beta subcomplex subunit 10 is one of the proteins that have the highest mimicry and it is located in the complex I of mitochondria. Viruses may use mitochondrial energy pathways for their own bioenergetic and biosynthesis needs (Combs et al., 2020). Mitochondrial complex I activity is also linked with dementia in Parkinson’s disease (Gatt et al., 2016) and bipolar disorder (Callaly et al., 2015).

SARS-CoV-2 proteins interact with growth factors and their receptors. FGF1 is also one of the interactions of SARS-CoV-2. FGF family uses Ras/MAPK, PI3, and PLCγ pathways (Diez Del Corral and Morales, 2017), through which it helps for neuroprotection, cell survival, and neural differentiation during embryonic development, it is also linked with epileptogenesis, mood disorders and autoimmunity in the brain (Turner et al., 2016). Compared to other types of FGF, FGF 2 not only increases the cell survival, but they also help the maturation of the astrocytes and alter glutamate transport by the astrocytes through interaction with glutamate transporter 1 in mouse (Savchenko et al., 2019). By this effect, they contribute to neuroprotection in two ways, increasing cell survival and avoiding excitotoxicity induced by accumulated glutamate. EGFR is also a target of SARS-CoV-2 proteins. When ligands bind to EGFR receptors, they activate tyrosine kinase pathways and increase intracellular signaling through MAPK pathways (Novak et al., 2001). Other coronaviruses as MERS-COV and SARS-CoV were shown to interact with P38/MAPK and JAK-STAT pathways and their replication were inhibited when these pathways are inhibited (Lim et al., 2016). SARS-CoV virus was shown to activate both p38/MAPK, PI3, and JNK pathways in Vero E6 cells (Mizutani, 2007). JNK and p38/MAPK were upregulated in the transfected cells, whereas ERK and Akt were downregulated (Surjit et al., 2004; Mizutani, 2007). Through its downstream effects, it was suggested to take a role in both cell survival and apoptosis (Mizutani et al., 2004). JNK and PI3 pathways were also found to be important for the persistence of the virus in the cell (Mizutani, 2007). PI3 pathway is important for autophagy (Wong and Sanyal, 2020). SARS-CoV’s M protein-induced apoptosis through mitochondrial cytochrome c release (Chan et al., 2007). In vitro studies also proved the modulation of the ERK/MAPK and PI3K/AKT/mTOR pathways by MERS-COV for its replication (Kindrachuk et al., 2015). By affecting the autophagy pathways, mitochondrial dysfunction related to ROS production, increasing endoplasmic reticulum stress and protein misfolding, SARS-CoV-2 may increase vulnerability for neurodegeneration (Lippi et al., 2020).

The observed changes by the virus as increased chronic inflammation, mitochondria-related increased reactive oxygen species, and neurodegeneration, in addition to alteration in growth factors and second messenger system and neurodevelopmental effects on axonal guidance and neuronal growth, are shared mechanisms in the neurobiology of autism, schizophrenia, bipolar disorder and epilepsy (Konradi et al., 2012). Maternal infections are also known to increase the vulnerability of neurodevelopmental disorders, and its mechanism is not solely dependent on the mother’s fever and high inflammatory responses (Simanek and Meier, 2015; Flinkkila et al., 2016; Al-Haddad et al., 2019; Gustavson et al., 2019). Persistent viral infection and epitope spreading may cause chronic inflammation and neurodegeneration (Smatti et al., 2019). Placental passage of viruses, persistence in the brain, and mild chronic inflammation are also discussed for the development of psychiatric disorders. Although in 9 newborn from COVID-19 positive mothers were negative (amniotic fluid, cord blood, neonatal throat swab tested), another pathway of transmission in addition to respiration and entry through the mouth and nose, the placental passage of SARS-CoV-2 (Dong et al., 2020) is also discussed in a case report where the nasal swab of the newborn was negative but IgM levels were high and newborn was showing COVID-19 symptoms. Therefore, intrauterine infection of the fetus and its neurological and psychiatric outcomes are also questioned. As another neuroinvasive virus, HIV-1 may produce a protected viral reservoir in the brain and neuropsychiatric manifestations are thought to result from the direct cytopathic effects of the virus (Schroecksnadel et al., 2012). We found that SARS-CoV-2 proteins also significantly interact with axon guidance molecules, neuronal growth, and survival. In the case of a placental passage and latent infection in the brain, SARS-CoV-2 could also be linked to neurodevelopmental disorders. As an example, genetic variations in SNAP-29 are also linked with neurodevelopment disorders as observed in 22q11.2 deletion syndrome (di George syndrome) which is linked with schizophrenia and bipolar disorder (Kadkova et al., 2019).

Other than the direct effects on the brain, SARS-CoV-2 may indirectly affect the brain. From clinical cases, it is well defined that thrombosis formation and vascular occlusion are troublesome for especially patients in ICU and going through ventilation-intubation. In a recent article, the authors described the pathophysiology of SARS-CoV-2 induced stroke in a pericyte based approach. They have demonstrated that, in CNS, BBB pericytes are the only cells with high expression of ACE-2 receptors and endothelial cells do not express ACE-2 receptors at all (He et al., 2020). They hypothesize that in the elderly, especially having diabetes mellitus and hypertension, endothelia become leaky, leading to easy passage of viruses (He et al., 2020). From our analysis on coagulation factors, it is evident that the virus can target coagulation pathways in multiple ways like affecting either blood coagulation factors as prothrombin, fibrinogen, coagulation factors VII, X, XI and von Willebrand factor or inhibition of coagulation like tissue plasminogen activator, plasminogen activator inhibitor. Additionally, we have detected that several viral particles may interact with subunits of hemoglobin. These similarities may explain decreased tissue oxygenation due to viral interference if this interaction leads to the decreased oxygen-binding capacity of hemoglobin. Interaction of viral proteins with fibrinogen in plasma may lead to activation of fibrinogen leading to fibrin polymer generation hence coagulation. On top of it, it may interact with factor VII, anti-thrombin, and other our listed coagulation related proteins leading to either loss of function or gain of function. Hence, it is not surprising that coagulopathy is one of the hallmark clinical presentations. Since here we describe computationally based similarities, all the proposed interactions must be studied in wet lab setups to establish direct effects of interactions.

In contrast to direct invasion and vascular reactions, most of the neurological involvement of viral-induced diseases may have CNS consequences due to systemic inflammatory conditions. This is mostly due to inflammatory responses induced by virus changes in BBB and a vicious circle leading to an inflammatory response in immune-privileged brain tissue like lymphocyte, neutrophil passage to brain, and induction of microglia-macrophage response of CNS. Systemic inflammatory molecules like IL-6 may have these effects but especially some interleukins might be involved, especially in CNS. From the list of proteins taking part in inflammation, IL-21, IL-22, IL-1B are important proteins that are involved in MS, Parkinson’s disease, and other viral-bacterial CNS infections (Wang et al., 2012; Perriard et al., 2015; Xin et al., 2015). TGF-beta is known as an inducer of inflammation and vascular reaction in BBB and it is a strong stimulator of pericytes and macrophages (Rustenhoven et al., 2016) and may lead to pericyte to myofibroblast transformation and fibrosis generation in other organs.

The mimicry of human protein interactions by SARS-CoV-2 may also be linked to the development of autoimmune disorders. The emergence of autoimmune disorders affecting distinct tissues has been reported and the immune response produced to cope with SARS-CoV-2 may interact with human proteins due to molecular mimicry (Rodriguez et al., 2020). Latent viral infections of the long-living neurons may cause its spread to other glial cells like astrocytes and oligodendrocytes and coronavirus infections in rats and cats are linked with autoimmune demyelinating disorders (Cohen, 2020). Many other viruses as measles, Epstein-Barr, and HTLV-1 have been linked with the emergence of autoimmune disorders like MS and this has been explained by bystander activation and molecular mimicry (Getts et al., 2013).

Additionally, we have detected that Calcitonin gene-related peptide type 1 receptor may have an interaction with spike protein. It is also interesting that this protein has a role in headache generation as well as gastrointestinal functions. Hence, if there is possible interaction, this may explain headache and some of the gastrointestinal symptoms of COVID-19.

Lastly, SARS-CoV-2 also targets insulin pathways and GLP-1 related regulation of blood glucose control. Literature supports this finding by reporting a 37-year-old previously healthy man with normal BMI that was diagnosed with diabetic ketoacidosis at the time of COVID-19 diagnosis (Chee et al., 2020). Another 54-year-old man with no diabetic history also was reported to present to the emergency room with hyperglycemia and shortness of breath and diagnosed COVID-19 (Heaney et al., 2020). COVID-19 diagnosed patients also presented with non-diabetic ketoacidosis in addition to the worsening of diabetic ketoacidosis (Kim N. Y. et al., 2020; Li J. et al., 2020). Diabetes is also known to increase the mortality of COVID-19 cases (Huang et al., 2020). The DPP-4 molecule was also used by MERS-COV as a receptor (Iwata-Yoshikawa et al., 2019; Widagdo et al., 2019). These molecules not only take part in glucose regulation, but they may have both indirect and direct effects in the brain, as especially GLP-1 has its receptors in the brain and it is related to neuroprotection (Erbil et al., 2019) and reward processing (Yapici-Eser et al., 2020).

This study has several limitations. First of all, our analysis is based on a computational model, therefore it may represent some false-positive results and the current possible interactions need to be assessed in cell culture and animal models for an in vivo interaction’s existence and further biological effects. Secondly, our analysis involves only 11 of the 29 proteins of SARS-CoV-2, as these are the only proteins whose protein structures are known. The involvement of other SARS-CoV-2 proteins in the analysis may change the statistical overrepresentation of involved pathways. The pathways associated with the interaction list and subcategory lists have been generated using PANTHER classification, in addition to authors’ knowledge and literature search. There may still be some proteins missed for inclusion in the Supplementary Tables 5–12. This study needs to be supported by clinical studies with large sample sizes and longer follow up durations to observe a wider outcome of the SARS-CoV-2 mimicry of human interactions on the nervous system.

In conclusion, we represent here the candidate proteins that are most likely to affect the neuropsychiatric representations of COVID-19. SARS-CoV-2 proteins mimic the human protein interactions for blood-brain barrier formation, synaptic vesicle trafficking, endocytosis, axonal transport, neurotransmission, apoptosis and also coagulation, inflammation, and metabolic control, which may result in the development of delirium, psychotic features, seizures, encephalitis, stroke, sensory impairments, peripheric nerve diseases, and autoimmune disorders. With different personal risk factors, different pathways may be triggered, and may cause a different phenotype for COVID-19 clinical presentation. Our findings are also supported by the previous in vivo and in vitro studies from other viruses. Similar studies can be conducted to better understand SARS-CoV-2 pathology. Further in vivo and in vitro studies using the proteins that we pointed to, could pave new targets both for avoiding and reversing neuropsychiatric presentations.

## Conclusion

SARS-CoV-2 proteins mimic the human protein-protein interactions. Among the human proteins mimicked by SARS-CoV-2 proteins, many are linked to synaptic vesicle trafficking, endocytosis, axonal transport, neurotransmission, growth factors, mitochondrial and blood-brain barrier elements, in addition to its peripheral interactions with proteins linked to thrombosis, inflammation and metabolic control. Mimicry of human protein interactions by SARS-CoV-2 may explain the development of delirium, psychosis, seizures, encephalitis, stroke, sensory impairments, peripheral nerve diseases, and autoimmune disorders.

## Author Contributions

HY-E, OK, AG, and YG-O designed and conceived the study. YK prepared the HMI-PRED protein interaction data. HY-E conducted the PANTHER analysis. HY-E, YG-O, and OO-C generated the supplementary lists of proteins distributed for functions. All authors contributed significantly to writing the draft and approved the final version of the manuscript.

## Conflict of Interest

The authors declare that the research was conducted in the absence of any commercial or financial relationships that could be construed as a potential conflict of interest.

## Footnotes

1 https://www.who.int/emergencies/diseases/novel-coronavirus-2019

2 http://www.pantherdb.org/

3 http://www.rosettacommons.org
