# Supplementary material for: Neuropsychiatric Symptoms of COVID-19 Explained by SARS-CoV-2 Proteins’ Mimicry of Human Protein Interactions
Source: Front Hum Neurosci. 2021 Mar 23;15:656313. doi: 10.3389/fnhum.2021.656313 (PMC8021734; doi:10.3389/fnhum.2021.656313)
Supplement: Supplementary file 1 [file Data_Sheet_1.docx]

**Supplemental Information titles and legends**

**Supplementary Table 1.** SARS-CoV-2 proteins, their functions, and available structures in the Protein Data Bank (PDB) with respective PDB IDs. Final PDBs taken on May 9.

**Supplementary Table 2.** List of SARS-CoV-2 proteins’ mimicry of human protein interactions.

**Supplementary Table 3.** PANTHER pathways associated with SARS-CoV-2 proteins’ mimicry of human protein interactions.

**Supplementary Table 4.** PANTHER Pathways statistically overrepresented in the distribution of proteins in pathways of SARS-CoV-2 proteins’ mimicry of human protein interactions.

**Supplementary Table 5.** SARS-CoV-2 proteins mimicking the human protein interactions in synaptic vesicle trafficking, vesicle fusion, and endocytosis

**Supplementary Table 6.** SARS-CoV-2 proteins mimicking the human protein interactions for neurotransmission

**Supplementary Table 7.** SARS-CoV-2 proteins mimicking the human protein interactions for growth factors

**Supplementary Table 8.** SARS-CoV-2 proteins mimicking the human protein interactions for mitochondria

**Supplementary Table 9.** SARS-CoV-2 proteins mimicking the human protein interactions for axonal guidance

**Supplementary Table 10.** SARS-CoV-2 proteins mimicking the human protein interactions for blood-brain barrier

**Supplementary Table 11.** SARS-CoV-2 proteins mimicking the human protein interactions for coagulation

**Supplementary Table 12.** SARS-CoV-2 proteins mimicking the human protein interactions for inflammation

#### Supplementary Table 1. SARS-CoV-2 proteins, their functions, and available structures in the Protein Data Bank (PDB) with respective PDB IDs. Final PDBs taken on May 9.

| Name of protein | Function of the protein | Structures in PDB |
| --- | --- | --- |
| Nsp1 | Inhibits host gene expression and degrades host mRNA | no available structure |
|  |  |  |
| Nsp2 | Interacts with PHB1 and PHB2 to alter host cell environment | no available structure |
|  |  |  |
| Nsp3 (Papain-like Protease) | Responsible for viral replication, forms NSP3-NSP4-NSP6 complex | 6W02,6W6Y,6VXS,6WEN,6WCF,6W9C |
|  |  |  |
| Nsp4 | Possible membrane arrangement function, forms NSP3-NSP4-NSP6 complex | no available structure |
|  |  |  |
| Nsp5 | 3CL-like (Main) Protease, functions in maturation of NSP protein via cleavage | 6Y2G,6Y2F,6Y2E,6Y84,6W63,6YB7,5R84,5R83,5R7Y,5R80,5R82,5R81,5R7Z,5REA,5REC,5REB,5REE,5RED,5REG,5REF,5RE9,5RE8,5RE5,5RE4,5RE7,5RE6,5RFB,5RFA,5RFD,5RFC,5RFF,5RFE,5RFH,5RFG,5REY,5REX,5RF9,5REZ,5RF2,5REP,5RF1,5RES,5RF4,5RER,5RF3,5REU,5RF6,5RET,5RF5,5REW,5RF8,5REV,5RF7,5REI,5REH,5REK,5REJ,5REM,5REL,5REO,5RF0,5REN,5RFZ,5RFY,5RFR,5RFQ,5RFT,5RFS,5RFV,5RFU,5RFX,5RFW,5RFJ,5RFI,5RFL,5RFK,5RFN,5RFM,5RFP,5RFO,5RG0,6M03,6LU7,5R8T,5RG1,5RG2,5RG3,5RGG,5RGH,5RGI,5RGJ,5RGK,5RGL,5RGM,5RGN,5RGO,5RGP,5RGQ,5RGR,5RGS,6M2N,6M2Q,7BQY |
|  |  |  |
| Nsp6 | Forms NSP3-NSP4-NSP6 complex | no available structure |
|  |  |  |
| Nsp7 | Forms NSP7-NSP8 complex which join RNA Polymerase (NSP12) | 6M71,7BTF,6WIQ,7BV2,7BV1,6WIQ |
|  |  |  |
| Nsp8 | Forms NSP8-NSP12 complex to form RNA Polymease complex | 6M71,7BTF,7BV2,7BV1 |
|  |  |  |
| Nsp9 | Binds ssRNAs, possible function in viral replication | 6W4B,6W9Q |
|  |  |  |
| Nsp10 | Interacts with NSP14, Stimulates methyl transferase activity | 6W61, 6W4H, 6W75, 6WJT |
|  |  |  |
| Nsp11 | Short peptide | no available structure |
|  |  |  |
| Nsp12 | RNA polymerase, copies viral RNA, increase helicase activity, forms NSP7-NSP8-NSP12 complex | 6M71,7BTF,7BV2,7BV1 |
|  |  |  |
| Nsp13 | RNA Helicase, unwinds duplex RNA, functions as triphosphate | no available structure |
|  |  |  |
| Nsp14 | Functions as Exoribonuclease, N7-methyltransferase | no available structure |
|  |  |  |
| Nsp15 | Functions as Endoribonuclease, targets RNA uridines | 6W01,6VWW |
|  |  |  |
| Nsp16 | 2'-O-methyltransferase, functions in mRNA translation, provides virus some degree of immunity | 6W61,6W4H,6W75,6WJT |
|  |  |  |
| S | Spike, recognition and binding of the virus to the host cell | 6LVN,6LXT,6LZG,6YLA,6VW1,6M17,6M0J,6W41,6VYB,6VXX,6VSB |
|  |  |  |
| E | Envelope, integral membrane protein which oligomerize and create an ion channel | no available structure |
|  |  |  |
| M | Membrane glycoprotein, main component of the envelope, functions in viral assembly | no available structure |
|  |  |  |
| N | Nucleocapsid phosphoprotein, binds viral RNA for stability | 6WJI,6VYO,6M3M,6YI3 |
|  |  |  |
| Orf3a | Ion channel, related with NLRP3 inflammasome | no available structure |
|  |  |  |
| Orf3b | No information | no available structure |
|  |  |  |
| Orf6 | Possible function in viral-induced apoptosis, Type I IFN antagonist | no available structure |
|  |  |  |
| Orf7a | Transmembrane protein, related to virus-induced apoptosis | no available structure |
|  |  |  |
| Orf7b | No information | no available structure |
|  |  |  |
| Orf8 | No information | no available structure |
|  |  |  |
| Orf9b | Type I IFN antagonist | no available structure |
|  |  |  |
| Orf9c | No information | no available structure |
|  |  |  |
| Orf10 | No information | no available structure |

#### Supplementary Table 2. List of SARS-CoV-2 proteins’ mimicry of human protein interactions.

#### <http://prism.ccbb.ku.edu.tr/data/hmisars/>

#### Supplementary Table 3. PANTHER pathways associated with SARS-CoV-2 proteins’ mimicry of human protein interactions.

| Pathway Name and Panther Pathway Code | Number of proteins |
| --- | --- |
| Gonadotropin-releasing hormone receptor pathway (P06664) | 89 |
| CCKR signaling map (P06959) | 77 |
| Inflammation mediated by chemokine and cytokine signaling pathway (P00031) | 68 |
| Integrin signalling pathway (P00034) | 63 |
| Angiogenesis (P00005) | 63 |
| Apoptosis signaling pathway (P00006) | 52 |
| Huntington disease (P00029) | 49 |
| EGF receptor signaling pathway (P00018) | 45 |
| PDGF signaling pathway (P00047) | 45 |
| T cell activation (P00053) | 45 |
| Parkinson disease (P00049) | 43 |
| Wnt signaling pathway (P00057) | 42 |
| TGF-beta signaling pathway (P00052) | 41 |
| FGF signaling pathway (P00021) | 39 |
| Interleukin signaling pathway (P00036) | 36 |
| p53 pathway (P00059) | 35 |
| Ras Pathway (P04393) | 34 |
| Ubiquitin proteasome pathway (P00060) | 33 |
| B cell activation (P00010) | 31 |
| VEGF signaling pathway (P00056) | 30 |
| p53 pathway feedback loops 2 (P04398) | 25 |
| Alzheimer disease-presenilin pathway (P00004) | 25 |
| Blood coagulation (P00011) | 24 |
| Heterotrimeric G-protein signaling pathway-Gi alpha and Gs alpha mediated pathway (P00026) | 24 |
| Toll receptor signaling pathway (P00054) | 24 |
| PI3 kinase pathway (P00048) | 22 |
| FAS signaling pathway (P00020) | 21 |
| Cytoskeletal regulation by Rho GTPase (P00016) | 20 |
| Transcription regulation by bZIP transcription factor (P00055) | 20 |
| Nicotinic acetylcholine receptor signaling pathway (P00044) | 18 |
| Heterotrimeric G-protein signaling pathway-Gq alpha and Go alpha mediated pathway (P00027) | 17 |
| Oxidative stress response (P00046) | 17 |
| Alzheimer disease-amyloid secretase pathway (P00003) | 17 |
| Cadherin signaling pathway (P00012) | 16 |
| Endothelin signaling pathway (P00019) | 16 |
| Insulin/IGF pathway-protein kinase B signaling cascade (P00033) | 16 |
| Metabotropic glutamate receptor group III pathway (P00039) | 16 |
| p38 MAPK pathway (P05918) | 15 |
| Insulin/IGF pathway-mitogen activated protein kinase kinase/MAP kinase cascade (P00032) | 15 |
| Cell cycle (P00013) | 13 |
| DNA replication (P00017) | 13 |
| Dopamine receptor mediated signaling pathway (P05912) | 13 |
| General transcription regulation (P00023) | 13 |
| Hypoxia response via HIF activation (P00030) | 13 |
| p53 pathway by glucose deprivation (P04397) | 13 |
| Axon guidance mediated by semaphorins (P00007) | 13 |
| Axon guidance mediated by netrin (P00009) | 13 |
| 5HT2 type receptor mediated signaling pathway (P04374) | 12 |
| Oxytocin receptor mediated signaling pathway (P04391) | 12 |
| Interferon-gamma signaling pathway (P00035) | 12 |
| Muscarinic acetylcholine receptor 1 and 3 signaling pathway (P00042) | 12 |
| Muscarinic acetylcholine receptor 2 and 4 signaling pathway (P00043) | 12 |
| Nicotine pharmacodynamics pathway (P06587) | 11 |
| Metabotropic glutamate receptor group II pathway (P00040) | 11 |
| Axon guidance mediated by Slit/Robo (P00008) | 11 |
| Thyrotropin-releasing hormone receptor signaling pathway (P04394) | 10 |
| Ionotropic glutamate receptor pathway (P00037) | 10 |
| Notch signaling pathway (P00045) | 10 |
| Beta1 adrenergic receptor signaling pathway (P04377) | 9 |
| Beta2 adrenergic receptor signaling pathway (P04378) | 9 |
| Opioid proenkephalin pathway (P05915) | 9 |
| 5HT1 type receptor mediated signaling pathway (P04373) | 8 |
| Opioid prodynorphin pathway (P05916) | 8 |
| Hedgehog signaling pathway (P00025) | 8 |
| Opioid proopiomelanocortin pathway (P05917) | 8 |
| Plasminogen activating cascade (P00050) | 8 |
| Angiotensin II-stimulated signaling through G proteins and beta-arrestin (P05911) | 7 |
| Enkephalin release (P05913) | 7 |
| Glycolysis (P00024) | 7 |
| GABA-B receptor II signaling (P05731) | 7 |
| De novo purine biosynthesis (P02738) | 7 |
| 5HT4 type receptor mediated signaling pathway (P04376) | 6 |
| Beta3 adrenergic receptor signaling pathway (P04379) | 6 |
| Cortocotropin releasing factor receptor signaling pathway (P04380) | 6 |
| Histamine H1 receptor mediated signaling pathway (P04385) | 6 |
| JAK/STAT signaling pathway (P00038) | 6 |
| De novo pyrimidine deoxyribonucleotide biosynthesis (P02739) | 6 |
| Adrenaline and noradrenaline biosynthesis (P00001) | 6 |
| Pyruvate metabolism (P02772) | 5 |
| Heterotrimeric G-protein signaling pathway-rod outer segment phototransduction (P00028) | 5 |
| P53 pathway feedback loops 1 (P04392) | 5 |
| Endogenous cannabinoid signaling (P05730) | 5 |
| Metabotropic glutamate receptor group I pathway (P00041) | 5 |
| De novo pyrimidine ribonucleotides biosythesis (P02740) | 5 |
| Fructose galactose metabolism (P02744) | 5 |
| Alpha adrenergic receptor signaling pathway (P00002) | 5 |
| 5HT3 type receptor mediated signaling pathway (P04375) | 4 |
| Cholesterol biosynthesis (P00014) | 4 |
| Vitamin D metabolism and pathway (P04396) | 4 |
| 5-Hydroxytryptamine degredation (P04372) | 3 |
| Circadian clock system (P00015) | 3 |
| Pyrimidine Metabolism (P02771) | 3 |
| Histamine H2 receptor mediated signaling pathway (P04386) | 3 |
| Salvage pyrimidine deoxyribonucleotides (P02774) | 3 |
| Serine glycine biosynthesis (P02776) | 3 |
| Adenine and hypoxanthine salvage pathway (P02723) | 3 |
| Salvage pyrimidine ribonucleotides (P02775) | 2 |
| Nicotine degradation (P05914) | 2 |
| Succinate to proprionate conversion (P02777) | 2 |
| Vitamin B6 metabolism (P02787) | 2 |
| Xanthine and guanine salvage pathway (P02788) | 2 |
| Synaptic vesicle trafficking (P05734) | 2 |
| TCA cycle (P00051) | 2 |
| Tetrahydrofolate biosynthesis (P02742) | 2 |
| Heme biosynthesis (P02746) | 2 |
| mRNA splicing (P00058) | 2 |
| Methylmalonyl pathway (P02755) | 2 |
| N-acetylglucosamine metabolism (P02756) | 2 |
| Ornithine degradation (P02758) | 2 |
| Pyridoxal-5-phosphate biosynthesis (P02759) | 2 |
| BMP/activin signaling pathway-drosophila (P06211) | 2 |
| GBB signaling pathway (P06214) | 2 |
| MYO signaling pathway (P06215) | 2 |
| 5-Hydroxytryptamine biosynthesis (P04371) | 1 |
| Pentose phosphate pathway (P02762) | 1 |
| Purine metabolism (P02769) | 1 |
| Pyridoxal phosphate salvage pathway (P02770) | 1 |
| S-adenosylmethionine biosynthesis (P02773) | 1 |
| Histamine synthesis (P04387) | 1 |
| General transcription by RNA polymerase I (P00022) | 1 |
| Sulfate assimilation (P02778) | 1 |
| Bupropion degradation (P05729) | 1 |
| Thiamin metabolism (P02780) | 1 |
| ATP synthesis (P02721) | 1 |
| Androgen/estrogene/progesterone biosynthesis (P02727) | 1 |
| Arginine biosynthesis (P02728) | 1 |
| Ascorbate degradation (P02729) | 1 |
| Coenzyme A biosynthesis (P02736) | 1 |
| Cysteine biosynthesis (P02737) | 1 |
| Formyltetrahydroformate biosynthesis (P02743) | 1 |
| Glutamine glutamate conversion (P02745) | 1 |
| ALP23B signaling pathway (P06209) | 1 |
| Mannose metabolism (P02752) | 1 |
| Methionine biosynthesis (P02753) | 1 |
| Methylcitrate cycle (P02754) | 1 |
| Activin beta signaling pathway (P06210) | 1 |
| DPP-SCW signaling pathway (P06212) | 1 |
| DPP signaling pathway (P06213) | 1 |
| SCW signaling pathway | 1 |
| Toll pathway-drosophila | 1 |

#### Supplementary Table 4. PANTHER Pathways statistically overrepresented in the distribution of proteins in pathways of SARS-CoV-2 proteins’ mimicry of human protein interactions.

| PANTHER Pathways | Homo sapiens - Reference List (20851) | Number of proteins in the interaction list | Expected number | Fold Enrichment | P-value |
| --- | --- | --- | --- | --- | --- |
| FAS signaling pathway (P00020) | 35 | 21 | 3.79 | 5.55 | 4E-06 |
| p53 pathway by glucose deprivation (P04397) | 22 | 13 | 2.38 | 5.46 | 2E-03 |
| Cell cycle (P00013) | 22 | 13 | 2.38 | 5.46 | 2E-03 |
| Ubiquitin proteasome pathway (P00060) | 59 | 33 | 6.38 | 5.17 | 2E-09 |
| Blood coagulation (P00011) | 46 | 24 | 4.98 | 4.82 | 3E-06 |
| Axon guidance mediated by semaphorins (P00007) | 25 | 13 | 2.7 | 4.81 | 6E-03 |
| T cell activation (P00053) | 88 | 45 | 9.52 | 4.73 | 4E-12 |
| p53 pathway feedback loops 2 (P04398) | 50 | 25 | 5.41 | 4.62 | 3E-06 |
| Insulin/IGF pathway-mitogen activated protein kinase kinase/MAP kinase cascade (P00032) | 31 | 15 | 3.35 | 4.47 | 3E-03 |
| Ras Pathway (P04393) | 74 | 34 | 8.01 | 4.25 | 5E-08 |
| Apoptosis signaling pathway (P00006) | 115 | 52 | 12.44 | 4.18 | 2E-12 |
| CCKR signaling map (P06959) | 172 | 77 | 18.61 | 4.14 | 1E-18 |
| B cell activation (P00010) | 70 | 31 | 7.57 | 4.09 | 6E-07 |
| VEGF signaling pathway (P00056) | 68 | 30 | 7.36 | 4.08 | 1E-06 |
| Parkinson disease (P00049) | 98 | 43 | 10.6 | 4.06 | 8E-10 |
| PI3 kinase pathway (P00048) | 52 | 22 | 5.63 | 3.91 | 2E-04 |
| Toll receptor signaling pathway (P00054) | 57 | 24 | 6.17 | 3.89 | 7E-05 |
| DNA replication (P00017) | 31 | 13 | 3.35 | 3.88 | 3E-02 |
| TGF-beta signaling pathway (P00052) | 98 | 41 | 10.6 | 3.87 | 8E-09 |
| Insulin/IGF pathway-protein kinase B signaling cascade (P00033) | 39 | 16 | 4.22 | 3.79 | 7E-03 |
| Interleukin signaling pathway (P00036) | 88 | 36 | 9.52 | 3.78 | 2E-07 |
| Hypoxia response via HIF activation (P00030) | 32 | 13 | 3.46 | 3.75 | 4E-02 |
| p53 pathway (P00059) | 87 | 35 | 9.41 | 3.72 | 5E-07 |
| Gonadotropin-releasing hormone receptor pathway (P06664) | 232 | 89 | 25.1 | 3.55 | 5E-18 |
| p38 MAPK pathway (P05918) | 40 | 15 | 4.33 | 3.47 | 3E-02 |
| Angiogenesis (P00005) | 172 | 63 | 18.61 | 3.39 | 9E-12 |
| Huntington disease (P00029) | 143 | 49 | 15.47 | 3.17 | 3E-08 |
| Transcription regulation by bZIP transcription factor (P00055) | 59 | 20 | 6.38 | 3.13 | 8E-03 |
| EGF receptor signaling pathway (P00018) | 136 | 45 | 14.71 | 3.06 | 5E-07 |
| Integrin signalling pathway (P00034) | 191 | 63 | 20.67 | 3.05 | 4E-10 |
| FGF signaling pathway (P00021) | 121 | 39 | 13.09 | 2.98 | 9E-06 |
| PDGF signaling pathway (P00047) | 145 | 45 | 15.69 | 2.87 | 3E-06 |
| Inflammation mediated by chemokine and cytokine signaling pathway (P00031) | 255 | 68 | 27.59 | 2.46 | 2E-07 |
|  |  |  |  |  |  |

#### Supplementary Table 5. SARS-CoV-2 proteins mimicking the human protein interactions in synaptic vesicle trafficking, vesicle fusion, and endocytosis*

| Human Target Code | Human Target Name | Interacting SARS-COV-2 protein |
| --- | --- | --- |
| AKAP5_HUMAN | A-kinase anchor protein 5 | NSP8 |
| AP2B1_HUMAN | AP-2 complex subunit beta | Spike, NSP7, Nucleocapsid |
| APBA3_HUMAN | Amyloid-beta A4 precursor protein-binding family A member 3 | Spike |
| CABIN_HUMAN | Calcineurin-binding protein cabin-1 | NSP7 |
| CANB1_HUMAN | Calcineurin subunit B type 1 | NSP7 |
| CLCN5_HUMAN | H(+)/Cl(-) exchange transporter 5 | NSP8 |
| DISC1_HUMAN | Disrupted in schizophrenia 1 protein | NSP12 |
| DLG2_HUMAN | Disks large homolog 2 | NSP12 |
| DLG4_HUMAN | Disks large homolog 4 | Spike, NSP8, NSP7, Papain-like Protease |
| DNM1L_HUMAN | Dynamin-1-like protein | NSP9, NSP8, 3-CL-like Protease |
| DRD2_HUMAN | D(2) dopamine receptor | NSP8, NSP7 |
| DYN1_HUMAN | Dynamin-1 | Spike, NSP10, NSP9, NSP8, NSP7, 3-CL-like Protease |
| DYN3_HUMAN | Dynamin-3 | NSP16 |
| EPHA1_HUMAN | Ephrin type-A receptor 1 | NSP10 |
| EPHA2_HUMAN | Ephrin type-A receptor 2 | NSP3, 3-CL-like Protease |
| EPHA4_HUMAN | Ephrin type-A receptor 4 | Spike |
| EPHA7_HUMAN | Ephrin type-A receptor 7 | NSP7 |
| EPHA8_HUMAN | Ephrin type-A receptor 8 | NSP8 |
| EPHB1_HUMAN | Ephrin type-B receptor 1 | Papain-like Protease |
| FAK1_HUMAN | Focal adhesion kinase 1 | Spike |
| FAK2_HUMAN | Protein-tyrosine kinase 2-beta | Spike, NSP8, NSP7 |
| FMR1_HUMAN | Synaptic functional regulator FMR1 | NSP16 |
| GBRA1_HUMAN | Gamma-aminobutyric acid receptor subunit alpha-1 | Spike |
| GBRG2_HUMAN | Gamma-aminobutyric acid receptor subunit gamma-2 | 3-CL-like Protease |
| GCYB1_HUMAN | Guanylate cyclase soluble subunit beta-1 | NSP9, NSP8, Nucleocapsid |
| GRIA2_HUMAN | Glutamate receptor 2 | NSP7 |
| GRM1_HUMAN | Metabotropic glutamate receptor 1 | NSP12, 3-CL-like Protease |
| GRM5_HUMAN | Metabotropic glutamate receptor 5 | NSP7 |
| GSK3B_HUMAN | Glycogen synthase kinase-3 beta | NSP15, NSP10 |
| HOME3_HUMAN | Homer protein homolog 3 | NSP8 |
| HSP7C_HUMAN | Heat shock cognate 71 kDa protein | Spike, NSP9, NSP7 |
| MX1_HUMAN | Interferon-induced GTP-binding protein Mx1 | NSP7 |
| MX2_HUMAN | Interferon-induced GTP-binding protein Mx2 | Spike, NSP8 |
| NLGNX_HUMAN | Neuroligin-4, X-linked | NSP15, NSP8 |
| NTF4_HUMAN | Neurotrophin-4 | Spike, NSP8, NSP7 |
| NUFP1_HUMAN | Nuclear fragile X mental retardation-interacting protein 1 | NSP8, NSP7 |
| PENK_HUMAN | Proenkephalin-A | NSP12, 3-CL-like Protease |
| PRS8_HUMAN | 26S proteasome regulatory subunit 8 | Spike, NSP15, 3-CL-like Protease |
| PSN1_HUMAN | Presenilin-1 | NSP8 |
| RAB10_HUMAN | Ras-related protein Rab-10 | NSP8 |
| RAB8A_HUMAN | Ras-related protein Rab-8A | Spike, NSP8, 3-CL-like Protease |
| SH3G2_HUMAN | Endophilin-A1 | Spike, NSP8, NSP7 |
| SNP25_HUMAN | Synaptosomal-associated protein 25 | Spike, NSP9, Nucleocapsid |
| SNP29_HUMAN | Synaptosomal-associated protein 29 | Spike, NSP7 |
| STX5_HUMAN | Syntaxin-5 | NSP8 |
| STX6_HUMAN | Syntaxin-6 | Spike, NSP9, NSP7 |
| SYCP1_HUMAN | Synaptonemal complex protein 1 | Spike, NSP7 |
| SYN3_HUMAN | Synapsin-3 | Spike |
| SYNJ1_HUMAN | Synaptojanin-1 | 3-CL-like Protease |
| SYTL2_HUMAN | Synaptotagmin-like protein 2 | Spike, NSP10 |
| UBP8_HUMAN | Ubiquitin carboxyl-terminal hydrolase 8 | NSP7, Papain-like Protease |
| VAMP2_HUMAN | Vesicle-associated membrane protein 2 | Spike, NSP8, Papain-like Protease |
| VAMP8_HUMAN | Vesicle-associated membrane protein 8 | Spike |

#### *: This list has been generated by merging synapse located proteins in the PANTHER classification, in addition to author’s selected proteins from the HMI-PRED list screening. For the TM scores and rosetta scores of each interaction, Supplementary Table 2 is referred.

#### Supplementary Table 6. SARS-CoV-2 proteins mimicking the human protein interactions for neurotransmission**

| Human Target Code | Human Target Name | Interacting SARS-COV-2 protein |
| --- | --- | --- |
| AA2AR_HUMAN | Adenosine receptor A2a | NSP7, 3-CL-like Protease, Nucleocapsid |
| ACES_HUMAN | Acetylcholinesterase | Spike |
| ACHA2_HUMAN | Neuronal acetylcholine receptor subunit alpha-2 | 3-CL-like Protease |
| ACHA4_HUMAN | Neuronal acetylcholine receptor subunit alpha-4 | Spike, NSP7, 3-CL-like Protease |
| ACHB2_HUMAN | Neuronal acetylcholine receptor subunit beta-2 | Spike, NSP7 |
| AL1A3_HUMAN | Aldehyde dehydrogenase family 1 member A3 | NSP10 |
| AL4A1_HUMAN | Delta-1-pyrroline-5-carboxylate dehydrogenase, mitochondrial | 3-CL-like Protease |
| AL7A1_HUMAN | Alpha-aminoadipic semialdehyde dehydrogenase | 3-CL-like Protease |
| ARHG1_HUMAN | Rho guanine nucleotide exchange factor 1 | NSP8, Nucleocapsid |
| CAC1C_HUMAN | Voltage-dependent L-type calcium channel subunit alpha-1C | Spike, NSP10, NSP7, 3-CL-like Protease |
| CAC1D_HUMAN | Voltage-dependent L-type calcium channel subunit alpha-1D | NSP8 |
| CALM1_HUMAN | Calmodulin-1 | Spike, NSP10, NSP9, NSP8, NSP7, 3-CL-like Protease |
| CALM2_HUMAN | Calmodulin-2 | Spike, NSP9, NSP8, NSP7, Papain-like Protease |
| CBP_HUMAN | CREB-binding protein | NSP8, NSP7 |
| CDK5_HUMAN | Cyclin-dependent-like kinase 5 | Spike, NSP10 |
| CHLE_HUMAN | Cholinesterase | 3-CL-like Protease |
| CNGA3_HUMAN | Cyclic nucleotide-gated cation channel alpha-3 | Spike, NSP10 |
| CREB1_HUMAN | Cyclic AMP-responsive element-binding protein 1 | Spike, NSP9, NSP7, 3-CL-like Protease |
| DCHS_HUMAN | Histidine decarboxylase | 3-CL-like Protease |
| DOPO_HUMAN | Dopamine beta-hydroxylase | NSP10 |
| DRD2_HUMAN | D(2) dopamine receptor | NSP8, NSP7 |
| EP300_HUMAN | Histone acetyltransferase p300 | Spike, NSP16,NSP10, NSP8, NSP7 |
| GABR1_HUMAN | Gamma-aminobutyric acid type B receptor subunit 1 | Spike, NSP7, Papain-like Protease, 3-CL-like Protease |
| GABR2_HUMAN | Gamma-aminobutyric acid type B receptor subunit 2 | Spike, NSP9, Papain-like Protease |
| GBB1_HUMAN | Guanine nucleotide-binding protein G(I)/G(S)/G(T) subunit beta-1 | Spike, NSP8 |
| GBG2_HUMAN | Guanine nucleotide-binding protein G(I)/G(S)/G(O) subunit gamma-2 | NSP8 |
| GBRA1_HUMAN | Gamma-aminobutyric acid receptor subunit alpha-1 | Spike |
| GBRB2_HUMAN | Gamma-aminobutyric acid receptor subunit beta-2 | Spike, 3-CL-like Protease |
| GBRB3_HUMAN | Gamma-aminobutyric acid receptor subunit beta-3 | Spike, NSP7, 3-CL-like Protease |
| GBRG2_HUMAN | Gamma-aminobutyric acid receptor subunit gamma-2 | 3-CL-like Protease |
| GCR_HUMAN | Glucocorticoid receptor | Spike, NSP7 |
| GLRA1_HUMAN | Glycine receptor subunit alpha-1 | Spike |
| GLRA3_HUMAN | Glycine receptor subunit alpha-3 | Spike, NSP7, 3-CL-like Protease |
| GNAI1_HUMAN | Guanine nucleotide-binding protein G(i) subunit alpha-1 | NSP7 |
| GNAI3_HUMAN | Guanine nucleotide-binding protein G(k) subunit alpha | NSP7 |
| GPSM2_HUMAN | G-protein-signaling modulator 2 | Spike, NSP8 |
| GRB2_HUMAN | Growth factor receptor-bound protein 2 | 3-CL-like Protease |
| GRIA2_HUMAN | Glutamate receptor 2 | NSP7 |
| GRM1_HUMAN | Metabotropic glutamate receptor 1 | NSP12, 3-CL-like Protease |
| GRM2_HUMAN | Metabotropic glutamate receptor 2 | NSP16 |
| GRM5_HUMAN | Metabotropic glutamate receptor 5 | NSP7 |
| GRM8_HUMAN | Metabotropic glutamate receptor 8 | Spike, NSP12, NSP8, NSP7 |
| GRP1_HUMAN | RAS guanyl-releasing protein 1 | Spike, Nucleocapsid |
| GSK3B_HUMAN | Glycogen synthase kinase-3 beta | NSP15, NSP10 |
| HVCN1_HUMAN | Voltage-gated hydrogen channel 1 | Spike |
| KAP1_HUMAN | cAMP-dependent protein kinase type I-beta regulatory subunit | NSP8 |
| KAP2_HUMAN | cAMP-dependent protein kinase type II-alpha regulatory subunit | Spike |
| KCC2A_HUMAN | Calcium/calmodulin-dependent protein kinase type II subunit alpha | Nucleocapsid |
| KCC2D_HUMAN | Calcium/calmodulin-dependent protein kinase type II subunit delta | NSP16, NSP8, NSP7,Papain-like Protease, 3- CL-like Protease |
| KCJ11_HUMAN | ATP-sensitive inward rectifier potassium channel 11 | NSP8, NSP7 |
| KCNH1_HUMAN | Potassium voltage-gated channel subfamily H member 1 | NSP8 |
| KCNN4_HUMAN | Intermediate conductance calcium-activated potassium channel protein 4 | Spike, NSP12, ,NSP9 |
| KCNQ1_HUMAN | Potassium voltage-gated channel subfamily KQT member 1 | Spike, NSP8, NSP7 |
| KCNQ2_HUMAN | Potassium voltage-gated channel subfamily KQT member 2 | NSP8 |
| KCNQ4_HUMAN | Potassium voltage-gated channel subfamily KQT member 4 | NSP8 |
| KPCG_HUMAN | Protein kinase C gamma type | 3-CL-like Protease |
| MCR_HUMAN | Mineralocorticoid receptor | NSP8 |
| MTOR_HUMAN | Serine/threonine-protein kinase mTOR | Spike, NSP8, NSP7 |
| NMDE1_HUMAN | Glutamate receptor ionotropic, NMDA 2A | NSP9 |
| NMDZ1_HUMAN | Glutamate receptor ionotropic, NMDA 1 | NSP8 |
| NNMT_HUMAN | Nicotinamide N-methyltransferase | Spike |
| PENK_HUMAN | Proenkephalin-A | NSP12, 3- CL-like Protease |
| PHKG2_HUMAN | Phosphorylase b kinase gamma catalytic chain, liver/testis isoform | NSP16, 3- CL-like Protease |
| PLCE1_HUMAN | 1-phosphatidylinositol 4,5-bisphosphate phosphodiesterase epsilon-1 | 3-CL-like Protease |
| PLCG1_HUMAN | 1-phosphatidylinositol 4,5-bisphosphate phosphodiesterase gamma-1 | NSP8, NSP7, 3- CL-like Protease |
| PLCG2_HUMAN | 1-phosphatidylinositol 4,5-bisphosphate phosphodiesterase gamma-2 | NSP16, NSP8, 3-CL-like Protease |
| PYGL_HUMAN | Glycogen phosphorylase, liver form | 3-CL-like Protease |
| RAP1A_HUMAN | Ras-related protein Rap-1A | NSP7 |
| RAP1B_HUMAN | Ras-related protein Rap-1b | NSP7, Papain-like Protease |
| RGS16_HUMAN | Regulator of G-protein signaling 16 | Spike, NSP7 |
| RHOA_HUMAN | Transforming protein RhoA | Spike, NSP10, NSP8, Papain-like Protease |
| RPGP1_HUMAN | Rap1 GTPase-activating protein 1 | 3-CL-like Protease |
| SCN5A_HUMAN | Sodium channel protein type 5 subunit alpha | NSP12, NSP9, NSP7, 3-CL-like Protease |
| SYUA_HUMAN | Alpha-synuclein | NSP7, 3-CL-like Protease |
| TPH2_HUMAN | Tryptophan 5-hydroxylase 2 | Spike |
| TRPM4_HUMAN | Transient receptor potential cation channel subfamily M member 4 | Spike, NSP7 |
| TY3H_HUMAN | Tyrosine 3-monooxygenase | NSP9, NSP7 |
| VDAC1_HUMAN | Voltage-dependent anion-selective channel protein 1 | Spike, NSP8, NSP7 |

#### **: This list has been generated by merging the proteins listed under the subcategories of neurotransmission related pathways presented in Supplementary Table3, in addition to author’s selected proteins from the HMI-PRED list screening. For the TM scores and rosetta scores of each interaction, Supplementary Table 2 is referred.

#### Supplementary Table 7. SARS-CoV-2 proteins mimicking the human protein interactions for growth factors^#^

| Human Target Code | Human Target Name | Interacting SARS-COV-2 protein |
| --- | --- | --- |
| CNTF_HUMAN | Ciliary neurotrophic factor | Spike, NSP9, NSP8, NSP7, NSP3 |
| EGF_HUMAN | Pro-epidermal growth factor | Spike, NSP7 |
| EGFR_HUMAN | Epidermal growth factor receptor | Spike, NSP9, NSP8, NSP7, 3-CL-like Protease, Nucleocapsid |
| ERR3_HUMAN | Estrogen-related receptor gamma | Spike, NSP9, NSP8, 3-CL-like Protease |
| ESR1_HUMAN | Estrogen receptor | Spike, NSP3, 3-CL-like Protease |
| ESR2_HUMAN | Estrogen receptor beta | 3-CL-like Protease |
| FGF1_HUMAN | Fibroblast growth factor 1 | NSP10, NSP8 |
| FGF13_HUMAN | Fibroblast growth factor 13 | NSP7 |
| FGF2_HUMAN | Fibroblast growth factor 2 | NSP8, NSP7 |
| FGFR1_HUMAN | Fibroblast growth factor receptor 1 | NSP15, NSP8, NSP7 3-CL-like Protease |
| FGFR2_HUMAN | Fibroblast growth factor receptor 2 | Spike, NSP12, NSP8, NSP7, Nucleocapsid |
| FGFR4_HUMAN | Fibroblast growth factor receptor 4 | NSP7 |
| GDNF_HUMAN | Glial cell line-derived neurotrophic factor | Spike, NSP7, NSP3 |
| GFRA2_HUMAN | GDNF family receptor alpha-2 | NSP8 |
| GFRAL_HUMAN | GDNF family receptor alpha-like | Spike, NSP15 |
| GHR_HUMAN | Growth hormone receptor | NSP8 |
| GLP1R_HUMAN | Glucagon-like peptide 1 receptor | NSP8, NSP7, NPS3 |
| GLUC_HUMAN | Glucagon | 3-CL-like Protease |
| GRB10_HUMAN | Growth factor receptor-bound protein 10 | Spike, NSP12 |
| GRB14_HUMAN | Growth factor receptor-bound protein 14 | 3-CL-like Protease |
| GRB2_HUMAN | Growth factor receptor-bound protein 2 | 3-CL-like Protease |
| GSK3B_HUMAN | Glycogen synthase kinase-3 beta | NSP15, NSP10 |
| IBP1_HUMAN | Insulin-like growth factor-binding protein 1 | NSP8 |
| IBP4_HUMAN | Insulin-like growth factor-binding protein 4 | Spike, NSP12, NSP7 3-CL-like Protease |
| IDE_HUMAN | Insulin-degrading enzyme | Spike |
| IF2B1_HUMAN | Insulin-like growth factor 2 mRNA-binding protein 1 | NSP7 |
| IGF1_HUMAN | Insulin-like growth factor I | 3-CL-like Protease |
| IGF1R_HUMAN | Insulin-like growth factor 1 receptor | Spike, NSP9, NSP7, Nucleocapsid |
| INS_HUMAN | Insulin | Spike, NSP12, NSP10, NSP9, NSP8, NSP7, 3-CL-like Protease |
| INSL3_HUMAN | Insulin-like peptide 3 | Spike |
| INSL5_HUMAN | Insulin-like peptide5 | NSP8 |
| INSR_HUMAN | Insulin receptor | Spike, NSP9 |
| NTF4_HUMAN | Neurotrophin-4 | Spike, NSP7 |
| PRL_HUMAN | Prolactin | NSP16 |
| PRLR_HUMAN | Prolactin receptor | NSP15, NSP8 |
| THB_HUMAN | Thyroid hormone receptor beta | Spike, NSP7 |
| VEGFA_HUMAN | Vascular endothelial growth factor A | NSP7, Nucleocapsid |
| VGFR2_HUMAN | Vascular endothelial growth factor receptor 2 | Spike, NSP8, NSP7 |

#### #: This list has been generated by merging the proteins listed under the subcategories of growth factor related pathways presented in Supplementary Table3, in addition to author’s selected proteins from the HMI-PRED list screening. For the TM scores and rosetta scores of each interaction, Supplementary Table 2 is referred.

#### Supplementary Table 8. SARS-CoV-2 proteins mimicking the human protein interactions for mitochondria^##^

| Human Target Code | Human Target Name^*^ | Interacting SARS-COV-2 protein |
| --- | --- | --- |
| C11B2_HUMAN | Cytochrome P450 11B2, mitochondrial | NSP10, NSP7, 3-CL-like Protease, Nucleocapsid |
| CP2A6_HUMAN | Cytochrome P450 2A6 | 3-CL-like Protease |
| CY1_HUMAN | Cytochrome c1, heme protein, mitochondrial | Spike, NSP10, 3-CL-like Protease |
| DHE3_HUMAN | Glutamate dehydrogenase 1, mitochondrial | Spike, NSP10, NSP8, NSP7 |
| FRDA_HUMAN | Frataxin, mitochondrial | Spike, NSP9, NSP8, NSP7, Papain-like Protease, 3-CL-like Protease |
| GSTA4_HUMAN | Glutathione S-transferase A4 | Spike, NSP8, Nucleocapsid |
| MICU1_HUMAN | Calcium uptake protein 1, mitochondrial | Spike, NSP10, NSP8, Papain-like Protease |
| MID51_HUMAN | Mitochondrial dynamics protein MID51 | Spike, NSP9, NSP7, Papain-like Protease |
| MRPP3_HUMAN | Mitochondrial ribonuclease P catalytic subunit | NSP7, Nucleocapsid |
| MTOR_HUMAN | Serine/threonine-protein kinase mTOR | Spike, NSP8, NSP7 |
| NDUA1_HUMAN | NADH dehydrogenase [ubiquinone] 1 alpha subcomplex subunit 1 | NSP7 |
| NDUA3_HUMAN | NADH dehydrogenase [ubiquinone] 1 alpha subcomplex subunit 3 | Nucleocapsid |
| NDUA5_HUMAN | NADH dehydrogenase [ubiquinone] 1 alpha subcomplex subunit 5 | Spike, NSP8, NSP7, 3-CL-like Protease, Nucleocapsid |
| NDUAB_HUMAN | NADH dehydrogenase [ubiquinone] 1 alpha subcomplex subunit 11 | Papain-like Protease |
| NDUB1_HUMAN | NADH dehydrogenase [ubiquinone] 1 beta subcomplex subunit 1 | Spike |
| NDUB3_HUMAN | NADH dehydrogenase [ubiquinone] 1 beta subcomplex subunit 3 | NSP8 |
| NDUB4_HUMAN | NADH dehydrogenase [ubiquinone] 1 beta subcomplex subunit 4 | Spike, NSP10, ,NSP8, Papain-like Protease |
| NDUB9_HUMAN | NADH dehydrogenase [ubiquinone] 1 beta subcomplex subunit 9 | Spike, NSP8, NSP7 |
| NDUBB_HUMAN | NADH dehydrogenase [ubiquinone] 1 beta subcomplex subunit 11, mitochondrial | Spike, NSP7 |
| NDUS4_HUMAN | NADH dehydrogenase [ubiquinone] iron-sulfur protein 4, mitochondrial | NSP16, Papain-like Protease, 3-CL- like Protease |
| ODO1_HUMAN | 2-oxoglutarate dehydrogenase, mitochondrial | Spike, NSP8, NSP7 |
| SIR5_HUMAN | NAD-dependent protein deacylase sirtuin-5, mitochondrial | Spike, NSP7, Papain-like Protease |
| SODC_HUMAN | Superoxide dismutase [Cu-Zn] | NSP9, NSP8, NSP7 |
| SODM_HUMAN | Superoxide dismutase [Mn], mitochondrial | NSP8, NSP7 |
| TIM10_HUMAN | Mitochondrial import inner membrane translocase subunit Tim10 | NSP8, NSP7 |
| TIM9_HUMAN | Mitochondrial import inner membrane translocase subunit Tim9 | Spike, NSP10, NSP8, NSP7, Nucleocapsid |
| TRAP1_HUMAN | Heat shock protein 75 kDa, mitochondrial | Spike, NSP8, NSP7, Papain-like Protease |
| UGDH_HUMAN | UDP-glucose 6-dehydrogenase | Spike, NSP8 |

#### ^##^: These proteins have been selected among the more than 240 mitochondrial proteins listed in Supplementary Table 2. For the TM scores and rosetta scores of each interaction, Supplementary Table 2 is referred.

#### Supplementary Table 9. SARS-CoV-2 proteins mimicking the human protein interactions for axonal guidance^###^

| Human Target Code | Human Target Name | Interacting SARS-COV-2 protein |
| --- | --- | --- |
| ABL1_HUMAN | Tyrosine-protein kinase ABL1 | Spike, NSP16, NSP7 |
| ARHG1_HUMAN | Rho guanine nucleotide exchange factor 1 | NSP8, Nucleocapsid |
| CDC42_HUMAN | Cell division control protein 42 homolog | Spike, NSP10, NSP8, NSP7, Papain-like Protease, 3-CL-like Protease |
| CDK5_HUMAN | Cyclin-dependent-like kinase 5 | Spike, NSP10 |
| CXCR4_HUMAN | C-X-C chemokine receptor type 4 | NSP8, Nucleocapsid |
| DCC_HUMAN | Netrin receptor DCC | Spike, NSP8 |
| DPYL1_HUMAN | Dihydropyrimidinase-related protein 1 | Spike, Papain-like Protease |
| DPYL3_HUMAN | Dihydropyrimidinase-related protein 3 | Spike, NSP10, NSP7, Papain-like Protease |
| DPYL5_HUMAN | Dihydropyrimidinase-related protein 5 | NSP12, Papain-like Protease |
| ENAH_HUMAN | Protein enabled homolog | Spike |
| FYN_HUMAN | Tyrosine-protein kinase Fyn | 3-CL-like Protease |
| NFAC2_HUMAN | Nuclear factor of activated T-cells, cytoplasmic 2 | NSP8 |
| NFAC3_HUMAN | Nuclear factor of activated T-cells, cytoplasmic 3 | NSP8 |
| OBSCN_HUMAN | Obscurin | NSP15 |
| P85A_HUMAN | Phosphatidylinositol 3-kinase regulatory subunit alpha | 3-CL-like Protease |
| PAK1_HUMAN | Serine/threonine-protein kinase PAK 1 | NSP7, Papain-like Protease, 3-CL-like Protease |
| PK3CD_HUMAN | Phosphatidylinositol 4,5-bisphosphate 3-kinase catalytic subunit delta isoform | NSP8, NSP7 |
| PLCG1_HUMAN | 1-phosphatidylinositol 4,5-bisphosphate phosphodiesterase gamma-1 | NSP8, NSP7, 3-CL-like Protease |
| PLCG2_HUMAN | 1-phosphatidylinositol 4,5-bisphosphate phosphodiesterase gamma-2 | NSP16, NSP8, 3-CL-like Protease |
| PLXB1_HUMAN | Plexin-B1 | Spike, Papain-like Protease |
| RAC1_HUMAN | Ras-related C3 botulinum toxin substrate 1 | Spike, NSP12, NSP8, NSP7 |
| RAC2_HUMAN | Ras-related C3 botulinum toxin substrate 2 | Spike, NSP7 |
| RAC3_HUMAN | Ras-related C3 botulinum toxin substrate 3 | NSP8 |
| RHOA_HUMAN | Transforming protein RhoA | Spike, NSP10, NSP8, Papain-like Protease |
| SDF1_HUMAN | Stromal cell-derived factor 1 | Spike, NSP8, NSP7 |
| SLIT2_HUMAN | Slit homolog 2 protein | NSP8, 3-CL-like Protease |

#### ###: This list includes the proteins listed under the three PANTHER pathways: axon guidance mediated by semaphorins (P00007), axon guidance mediated by netrin (P00009) and axon guidance mediated by Slit/Robo (P00008). For the TM scores and rosetta scores of each interaction, Supplementary Table 2 is referred.

|  |
| --- |

#### Supplementary Table 10. SARS-CoV-2 proteins mimicking the human protein interactions for blood-brain barrier ^#*^

| Human Target Code | Human Target Name | Interacting SARS-COV-2 protein |
| --- | --- | --- |
| ABCC8_HUMAN | ATP-binding cassette sub-family C member 8 | Spike, NSP8 |
| ABCG5_HUMAN | ATP-binding cassette sub-family G member 5 | Spike |
| ACE_HUMAN | Angiotensin-converting enzyme | Spike |
| ACE2_HUMAN | Angiotensin-converting enzyme 2 | Spike |
| ACTB_HUMAN | Actin, cytoplasmic 1 | NSP15, NSP8 |
| B2MG_HUMAN | Beta-2-microglobulin | Spike, NSP12, NSP8, NSP7, Nucleocapsid |
| C43BP_HUMAN | Collagen type IV alpha-3-binding protein | Spike, NSP9 |
| CO1A1_HUMAN | Collagen alpha-1(I) chain | Nucleocapsid, NSP7 |
| CO3A1_HUMAN | Collagen alpha-1(III) chain | Spike,NSP8,3-CL-like Protease |
| CO4A1_HUMAN | Collagen alpha-1(IV) chain | Spike, Papain-like Protease, 3- CL- like Protease |
| EPAS1_HUMAN | Endothelial PAS domain-containing protein 1 | Spike, NSP8 |
| FIBA_HUMAN | Fibrinogen alpha chain | Spike, NSP8, Nucleocapsid |
| GDNF_HUMAN | Glial cell line-derived neurotrophic factor | Spike, NSP7, Papain-like Protease |
| GFAP_HUMAN | Glial fibrillary acidic protein | Spike, NSP8 |
| GTR3_HUMAN | Solute carrier family 2, facilitated glucose transporter member 3 | Spike, NSP8, Papain-like Protease |
| IGF1R_HUMAN | Insulin-like growth factor 1 receptor | Spike, NPS9,NSP7, Nucleocapsid |
| IL6RA_HUMAN | Interleukin-6 receptor subunit alpha | Papain-like Protease, Nucleocapsid |
| IL6RB_HUMAN | Interleukin-6 receptor subunit beta | Spike, NSP10 |
| INSR_HUMAN | Insulin receptor | Spike, NSP9 |
| ITA2B_HUMAN | Integrin alpha-IIb | Spike, NSP7, Papain-like Protease |
| ITA4_HUMAN | Integrin alpha-4 | NSP7, Papain-like Protease |
| ITB4_HUMAN | Integrin beta-4 | NSP8 |
| ITBP1_HUMAN | Integrin beta-1-binding protein 1 | Spike, NSP8, NSP7, 3- CL- like Protease |
| LAMB1_HUMAN | Laminin subunit beta-1 | Spike |
| LAMC1_HUMAN | Laminin subunit gamma-1 | Spike |
| LDLR_HUMAN | Low-density lipoprotein receptor | Spike |
| NRP2_HUMAN | Neuropilin-2 | NSP8 |
| PDGFB_HUMAN | Platelet-derived growth factor subunit B | NSP8 |
| PGFRB_HUMAN | Platelet-derived growth factor receptor beta | NSP8, Papain-like Protease, Nucleocapsid |
| S100B_HUMAN | Protein S100-B | NSP7 |
| S10A4_HUMAN | Protein S100-A4 | Spike, NSP9, NSP8, NSP7 |
| S10A9_HUMAN | Protein S100-A9 | Spike, NSP10, NSP9, NSP8, NSP7 |
| SODM_HUMAN | Superoxide dismutase [Mn], mitochondrial | NSP8, NSP7 |
| TFR1_HUMAN | Transferrin receptor protein 1 | Spike, NSP8, NSP7, 3-CL-like Protease |
| TIE2_HUMAN | Angiopoietin-1 receptor | Spike |
| TIMP1_HUMAN | Metalloproteinase inhibitor 1 | NSP8 |
| TPA_HUMAN | Tissue-type plasminogen activator | NSP8 |
| VEGFA_HUMAN | Vascular endothelial growth factor A | NSP7, Nucleocapsid |
| VGFR2_HUMAN | Vascular endothelial growth factor receptor 2 | Spike, NSP8, NSP7 |
| VIME_HUMAN | Vimentin | Spike, NSP10, NSP9, NSP8, NSP7 |
| VIPR2_HUMAN | Vasoactive intestinal polypeptide receptor 2 | NSP8 |
| ZO1_HUMAN | Tight junction protein ZO-1 | Spike |

#### ^#*:^ This list includes 21 proteins chosen among 188 proteins listed under PANTHER ontology . For the TM scores and rosetta scores of each interaction, Supplementary Table 2 is referred.

#### Supplementary Table 11. SARS-CoV-2 proteins mimicking the human protein interactions for coagulation^#*#*^

| Human Target Code | Human Target Name | Interacting SARS-COV-2 protein |
| --- | --- | --- |
| ANT3_HUMAN | Antithrombin-III | 3-CL-like Protease |
| CXCL7_HUMAN | Platelet basic protein | Papain-like Protease, 3-CL-like Protease |
| FIBA_HUMAN | Fibrinogen alpha chain | Spike, NSP8, Nucleocapsid |
| FIBB_HUMAN | Fibrinogen beta chain | Spike, NSP7 |
| FIBG_HUMAN | Fibrinogen gamma chain | Spike, NSP9, NSP8, NSP7,Papain-like Protease, 3-CL-like Protease |
| FINC_HUMAN | Fibronectin | NSP15,NSP12, NSP9, NSP7 |
| FRIL_HUMAN | Ferritin light chain | Spike, NSP7, Papain-like Protease, |
| HBA_HUMAN | Hemoglobin subunit alpha | Spike, NSP16, NSP10, NSP9, NSP8, NSP7 |
| HBB_HUMAN | Hemoglobin subunit beta | Spike,NSP12, Papain-like Protease, |
| HBD_HUMAN | Hemoglobin subunit delta | NSP9,NSP8, NSP7 |
| HBE_HUMAN | Hemoglobin subunit epsilon | NSP9, NSP7 |
| HEBP2_HUMAN | Heme-binding protein 2 | NSP8, NSP7, Nucleocapsid |
| HEP2_HUMAN | Heparin cofactor 2 | Spike, NSP9, 3-CL-like Protease |
| LAMC1_HUMAN | Laminin subunit gamma-1 | Spike |
| PAI1_HUMAN | Plasminogen activator inhibitor 1 | NSP8,Papain-like Protease |
| PLMN_HUMAN | Plasminogen | NSP9 |
| THRB_HUMAN | Prothrombin | Spike, NSP16, NSP8 |
| TPA_HUMAN | Tissue-type plasminogen activator | NSP8 |
| VWF_HUMAN | von Willebrand factor | Spike, Papain-like Protease |

#### ^#*#*:^ This list includes 20 proteins chosen among 24 proteins listed for coagulation in PANTHER ontology . For the TM scores and rosetta scores of each interaction, Supplementary Table 2 is referred.

#### Supplementary Table 12. SARS-CoV-2 proteins mimicking the human protein interactions for inflammation^#**^

| Human Target Code | Human Target Name | Interacting SARS-COV-2 protein |
| --- | --- | --- |
| ACTB_HUMAN | Actin, cytoplasmic 1 | NSP15, NSP8 |
| ACVL1_HUMAN | Serine/threonine-protein kinase receptor R3 | Spike, NSP8, 3-CL-like Proteinase |
| ACVR1_HUMAN | Activin receptor type-1 | Spike |
| AKT3_HUMAN | RAC-gamma serine/threonine-protein kinase | NSP8, 3-CL-like Protease |
| AL5AP_HUMAN | Arachidonate 5-lipoxygenase-activating protein | Spike, NSP10,NSP8, NSP7 |
| ATF2_HUMAN | Cyclic AMP-dependent transcription factor ATF-2 | Spike, NSP7 |
| AVR2B_HUMAN | Activin receptor type-2B | Papain-like Protease, 3-CL-like Protease |
| BMP2_HUMAN | Bone morphogenetic protein 2 | Spike, 3-Cl-like Protease |
| BMP3_HUMAN | Bone morphogenetic protein 3 | Spike |
| BMP6_HUMAN | Bone morphogenetic protein 6 | Spike |
| BMPR2_HUMAN | Bone morphogenetic protein receptor type-2 | NSP7, 3-CL-like Protease |
| BRAF_HUMAN | Serine/threonine-protein kinase B-raf | NSP16, NSP12, NSP7, |
| C5AR1_HUMAN | C5a anaphylatoxin chemotactic receptor 1 | Spike |
| CASP9_HUMAN | Caspase-9 | Spike, NSP9, 3- CL-like Protease |
| CBP_HUMAN | CREB-binding protein | NSP8, NSP7 |
| CCL3_HUMAN | C-C motif chemokine 3 | NSP7, 3-CL-like Protease |
| CCL4_HUMAN | C-C motif chemokine 4 | NSP10,NSP8 |
| CCL7_HUMAN | C-C motif chemokine 7 | NSP8 |
| CCR9_HUMAN | C-C chemokine receptor type 9 | NSP7 |
| CDC42_HUMAN | Cell division control protein 42 homolog | Spike, NSP10, NSP8, NSP7, Papain-like Protease, 3-CL-like Protease |
| CITE2_HUMAN | Cbp/p300-interacting transactivator 2 | Spike, NSP8, NSP7 |
| COKA1_HUMAN | Collagen alpha-1(XX) chain | NSP9 |
| CRYAB_HUMAN | Alpha-crystallin B chain | Spike, NSP15, 3-CL-like Protease |
| CSKP_HUMAN | Peripheral plasma membrane protein CASK | Spike, NSP8 |
| CXCR4_HUMAN | C-X-C chemokine receptor type 4 | NSP8, Nucleocapsid |
| CXL10_HUMAN | C-X-C motif chemokine 10 | NSP8, Nucleocapsid |
| DCP1A_HUMAN | mRNA-decapping enzyme 1A | Spike, NSP9, NSP8, NSP7 |
| EP300_HUMAN | Histone acetyltransferase p300 | Spike, NSP16, NSP10,NSP8, NSP7 |
| ETS1_HUMAN | Protein C-ets-1 | Spike, Papain-like Protease |
| FAK1_HUMAN | Focal adhesion kinase 1 | Spike |
| FAK2_HUMAN | Protein-tyrosine kinase 2-beta | Spike, NSP8, NSP7 |
| FKB1A_HUMAN | Peptidyl-prolyl cis-trans isomerase FKBP1A | NSP7 |
| FKB1B_HUMAN | Peptidyl-prolyl cis-trans isomerase FKBP1B | 3-CL-like Protease |
| GBG2_HUMAN | Guanine nucleotide-binding protein G(I)/G(S)/G(O) subunit gamma-2 | NSP8 |
| GDF15_HUMAN | Growth/differentiation factor 15 | Spike, NSP8, Nucleocapsid |
| GDF2_HUMAN | Growth/differentiation factor 2 | NSP8 |
| GDF5_HUMAN | Growth/differentiation factor 5 | Spike |
| GDF8_HUMAN | Growth/differentiation factor 8 | NSP8 |
| GDNF_HUMAN | Glial cell line-derived neurotrophic factor | Spike, NSP7, Papain-like Protease |
| GNAI1_HUMAN | Guanine nucleotide-binding protein G(i) subunit alpha-1 | NSP7 |
| GNAI3_HUMAN | Guanine nucleotide-binding protein G(k) subunit alpha | NSP7 |
| GRB2_HUMAN | Growth factor receptor-bound protein 2 | 3-CL-like Protease |
| HIF1A_HUMAN | Hypoxia-inducible factor 1-alpha | Spike, NSP8, Nucleocapsid |
| HSPB1_HUMAN | Heat shock protein beta-1 | 3-CL-like Protease |
| I10R2_HUMAN | Interleukin-10 receptor subunit beta | NSP8 |
| IKKA_HUMAN | Inhibitor of nuclear factor kappa-B kinase subunit alpha | NSP10, NSP7 |
| IKKB_HUMAN | Inhibitor of nuclear factor kappa-B kinase subunit beta | NSP8, NSP7, Papain-like Protease,, 3-CL-like Protease |
| IL15_HUMAN | Interleukin-15 | Spike, NSP10,NSP8 NSP7,Papain-like Protease,, Nucleocapsid |
| IL1B_HUMAN | Interleukin-1 beta | Spike, Papain-like Protease |
| IL8_HUMAN | Interleukin-8 | NSP9 |
| INAR1_HUMAN | Interferon alpha/beta receptor 1 | NSP8, NSP7 |
| INHBA_HUMAN | Inhibin beta A chain | NSP12, NSP7 |
| ITA4_HUMAN | Integrin alpha-4 | NSP7, Papain-like Protease |
| ITAL_HUMAN | Integrin alpha-L | NSP7, Papain-like Protease |
| ITB1_HUMAN | Integrin beta-1 | NSP8, Papain-like Protease, 3-CL-like Protease |
| ITB2_HUMAN | Integrin beta-2 | Nucleocapsid |
| JAK2_HUMAN | Tyrosine-protein kinase JAK2 | NSP10, NSP8, NSP7, Papain-like Protease, 3-CL-like Protease |
| JUN_HUMAN | Transcription factor AP-1 | Spike, NSP7 |
| JUND_HUMAN | Transcription factor jun-D | Spike |
| KCC2A_HUMAN | Calcium/calmodulin-dependent protein kinase type II subunit alpha | Nucleocapsid |
| KCC2D_HUMAN | Calcium/calmodulin-dependent protein kinase type II subunit delta | NSP16,NSP8, NSP7, Papain-like Protease,, 3-CL-like Protease |
| KPCG_HUMAN | Protein kinase C gamma type | 3-CL-like Protease |
| LPXN_HUMAN | Leupaxin | NSP9, NSP8, NSP7 |
| LTC4S_HUMAN | Leukotriene C4 synthase | Spike, NSP10,NSP8,NSP7, Nucleocapsid, Papain-like Protease |
| MK01_HUMAN | Mitogen-activated protein kinase 1 | NSP7 |
| MK03_HUMAN | Mitogen-activated protein kinase 3 | 3-CL-like Protease |
| MK08_HUMAN | Mitogen-activated protein kinase 8 | Spike |
| MK09_HUMAN | Mitogen-activated protein kinase 9 | NSP9 |
| MK10_HUMAN | Mitogen-activated protein kinase 10 | Papain-like Protease, 3-CL-like Protease |
| MK14_HUMAN | Mitogen-activated protein kinase 14 | NSP16, NSP7, 3-CL- like Protease |
| MP2K1_HUMAN | Dual specificity mitogen-activated protein kinase kinase 1 | Spike, NSP8, NSP7 |
| MP2K2_HUMAN | Dual specificity mitogen-activated protein kinase kinase 2 | Papain-like Protease |
| MYH7_HUMAN | Myosin-7 | Spike |
| MYH9_HUMAN | Myosin-9 | Spike, NSP8, 3-CL-like Proteinase |
| MYLK_HUMAN | Myosin light chain kinase, smooth muscle | Spike, NSP8 |
| MYLK2_HUMAN | Myosin light chain kinase 2, skeletal/cardiac muscle | NSP8, Nucleocapsid |
| NFAC2_HUMAN | Nuclear factor of activated T-cells, cytoplasmic 2 | NSP8 |
| NFAC3_HUMAN | Nuclear factor of activated T-cells, cytoplasmic 3 | NSP8 |
| NOS3_HUMAN | Nitric oxide synthase, endothelial | Spike |
| P85A_HUMAN | Phosphatidylinositol 3-kinase regulatory subunit alpha | 3-CL-like Protease |
| PAK1_HUMAN | Serine/threonine-protein kinase PAK 1 | NSP7, Papain-like Protease, 3-CL-like Protease |
| PAK2_HUMAN | Serine/threonine-protein kinase PAK 2 | NSP8 |
| PAK4_HUMAN | Serine/threonine-protein kinase PAK 4 | Spike, NSP7, 3-CL-like Protease |
| PAXI_HUMAN | Paxillin | NSP8, NSP7, Papain-like Protease |
| PDPK1_HUMAN | 3-phosphoinositide-dependent protein kinase 1 | NSP7 |
| PK3C3_HUMAN | Phosphatidylinositol 3-kinase catalytic subunit type 3 | NSP16 |
| PK3CA_HUMAN | Phosphatidylinositol 4,5-bisphosphate 3-kinase catalytic subunit alpha isoform | Spike, NSP8 |
| PK3CD_HUMAN | Phosphatidylinositol 4,5-bisphosphate 3-kinase catalytic subunit delta isoform | NSP8, NSP7 |
| PLCE1_HUMAN | 1-phosphatidylinositol 4,5-bisphosphate phosphodiesterase epsilon-1 | 3-CL-like Protease |
| PLCG1_HUMAN | 1-phosphatidylinositol 4,5-bisphosphate phosphodiesterase gamma-1 | NSP8, NSP7, 3-CL-like Protease |
| PLCG2_HUMAN | 1-phosphatidylinositol 4,5-bisphosphate phosphodiesterase gamma-2 | NSP16, NSP8, 3-CL-like Protease |
| PREX1_HUMAN | Phosphatidylinositol 3,4,5-trisphosphate-dependent Rac exchanger 1 protein | Papain-like Protease |
| RAC1_HUMAN | Ras-related C3 botulinum toxin substrate 1 | Spike,NSP12, NSP8,NSP7 |
| RAC2_HUMAN | Ras-related C3 botulinum toxin substrate 2 | Spike, NSP7 |
| RAC3_HUMAN | Ras-related C3 botulinum toxin substrate 3 | NSP8 |
| RASH_HUMAN | GTPase HRas | Spike, NSP7, Nucleocapsid |
| RASK_HUMAN | GTPase KRas | NSP8, Papain-like Protease,, 3-CL-like Protease |
| RHOA_HUMAN | Transforming protein RhoA | Spike, NSP10,NSP8, Papain-like Protease |
| ROCK1_HUMAN | Rho-associated protein kinase 1 | Spike, NSP15, NSP9, NSP8, NSP7 |
| SHIP2_HUMAN | Phosphatidylinositol 3,4,5-trisphosphate 5-phosphatase 2 | 3-CL-like Protease |
| SKIL_HUMAN | Ski-like protein | Papain-like Protease, 3-CL-like Protease |
| SMAD1_HUMAN | Mothers against decapentaplegic homolog 1 | NSP8, NSP7,Papain-like Protease, 3-CL-like Protease, Nucleocapsid |
| SMAD2_HUMAN | Mothers against decapentaplegic homolog 2 | Spike, NSP8, NSP7, Papain-like Protease, 3-CL-like Protease |
| SMAD3_HUMAN | Mothers against decapentaplegic homolog 3 | NSP8, NSP7 |
| SMAD4_HUMAN | Mothers against decapentaplegic homolog 4 | Spike, NSP8, NSP7 |
| SMAD7_HUMAN | Mothers against decapentaplegic homolog 7 | NSP9 |
| SOCS4_HUMAN | Suppressor of cytokine signaling 4 | NSP12 |
| SOS1_HUMAN | Son of sevenless homolog 1 | Spike, NSP9, NSP7,Papain-like Protease |
| SPHK1_HUMAN | Sphingosine kinase 1 | Spike |
| STAT1_HUMAN | Signal transducer and activator of transcription 1-alpha/beta | Spike, NSP7 |
| TAB1_HUMAN | TGF-beta-activated kinase 1 and MAP3K7-binding protein 1 | NSP10, Papain-like Protease |
| TF65_HUMAN | Transcription factor p65 | 3-CL-like Protease |
| TGFB1_HUMAN | Transforming growth factor beta-1 proprotein | Spike, NSP10,NSP8, NSP7, 3-CL-like Protease |
| TGFB2_HUMAN | Transforming growth factor beta-2 proprotein | NSP7 |
| TGFB3_HUMAN | Transforming growth factor beta-3 proprotein | Spike |
| TNFB_HUMAN | Lymphotoxin-alpha | Spike, NSP7, 3-CL-like Protease |
| VEGFA_HUMAN | Vascular endothelial growth factor A | NSP7, Nucleocapsid |
| VGFR2_HUMAN | Vascular endothelial growth factor receptor 2 | Spike, NSP8, NSP7 |
| VWF_HUMAN | von Willebrand factor | Spike, Papain-like Protease, |
| X3CL1_HUMAN | Fractalkine | NSP8 |
| ZFYV9_HUMAN | Zinc finger FYVE domain-containing protein 9 | NSP7, Papain-like Protease, |

#### ^#**:^ This list includes proteins listed under inflammation mediated by chemokine and cytokine signaling pathways and T cell activation pathways under PANTHER ontology. For the TM scores and rosetta scores of each interaction, Supplementary Table 2 is referred.
